# Discovering markers of healthy aging: a prospective study in a Danish male birth cohort

**DOI:** 10.18632/aging.102151

**Published:** 2019-08-26

**Authors:** Kiyana Zarnani, Thomas E. Nichols, Fidel Alfaro-Almagro, Birgitte Fagerlund, Martin Lauritzen, Egill Rostrup, Stephen M. Smith

**Affiliations:** 1 Functional Imaging Unit, Department of Clinical Physiology, Nuclear Medicine and PET, Copenhagen University Hospital Rigshospitalet, Glostrup, Denmark; 2 Center for Healthy Aging, University of Copenhagen, Copenhagen, Denmark; 3 Department of Neuroscience, University of Copenhagen, Copenhagen, Denmark; 4 Department of Clinical Medicine, Glostrup, Denmark; 5 Center for Neuropsychiatric Schizophrenia Research, Mental Health Center, Glostrup, Denmark; 6 Department of Psychology, University of Copenhagen, Copenhagen, Denmark; 7 Wellcome Centre for Integrative Neuroimaging, Oxford Centre for Functional Magnetic Resonance Imaging of the Brain (FMRIB), Nuffield Department of Clinical Neurosciences, University of Oxford, Oxford, UK; 8 Oxford Big Data Institute, Li Ka Shing, Centre For Health Information and Discovery, Nuffield Department of Population Health University of Oxford, Oxford, UK; 9 Department of Statistics, University of Warwick, Coventry, UK

**Keywords:** neurocognitive function, brain structure, aging, magnetic resonance imaging, risk factors

## Abstract

There is a pressing need to identify markers of cognitive and neural decline in healthy late-midlife participants. We explored the relationship between cross-sectional structural brain-imaging derived phenotypes (IDPs) and cognitive ability, demographic, health and lifestyle factors (non-IDPs). Participants were recruited from the 1953 Danish Male Birth Cohort (N=193). Applying an extreme group design, members were selected in 2 groups based on cognitive change between IQ at age ~20y (IQ-20) and age ~57y (IQ-57). Subjects showing the highest (n=95) and lowest (n=98) change were selected (at age ~57) for assessments on multiple IDPs and non-IDPs. We investigated the relationship between 453 IDPs and 70 non-IDPs through pairwise correlation and multivariate canonical correlation analysis (CCA) models. Significant pairwise associations included positive associations between IQ-20 and gray-matter volume of the temporal pole. CCA identified a richer pattern - a single “positive-negative” mode of population co-variation coupling individual cross-subject variations in IDPs to an extensive range of non-IDP measures (*r* = 0.75, P_corrected_ < 0.01). Specifically, this mode linked higher cognitive performance, positive early-life social factors, and mental health to a larger brain volume of several brain structures, overall volume, and microstructural properties of some white matter tracts. Interestingly, both statistical models identified IQ-20 and gray-matter volume of the temporal pole as important contributors to the inter-individual variation observed. The converging patterns provide novel insight into the importance of early adulthood intelligence as a significant marker of late-midlife neural decline and motivates additional study.

## Introduction

In countries with advanced economies, changes in age distributions, largely due to lower birth rates and increased life expectancy, has meant that the world’s population is increasingly older, with the number of persons over 80 projected to triple by 2050 [1]. Paradoxically, the success and opportunities presented by this new longevity has often been over-shadowed by the many challenges that come with a “top heavy” society. Specifically, the growing number of older adults have created an unprecedented demand on health care services, with increased vulnerability to cognitive decline, appreciable loss of autonomy, and need for institutional care threatening the economic security of families, communities and countries. With an estimated 47 million people currently diagnosed with dementia worldwide [2,3], the necessity of responding to these challenges are vital. In view of this, what was once referred to as ‘the elephant in the room’ [4] – i.e., the role of aging in cognitive decline – has now transpired into a scientifically challenging and compelling pursuit, the search to identify the constituents of a healthy brain and mind across the human lifespan [5–11].

Given the complexity of the aging process, at present there is no gold standard selection of age-related markers for assessing cognitive decline and disease progression in older adults [6–9,11–18]. Furthermore, with considerable individual variability observed in aging trajectories, identifying the various factors that may underlie this individualism has not been a trivial task [19]. Prior studies investigating lifespan influences on non-pathological aging have identified childhood IQ, socioeconomic position (SEP), and genetic markers as some of the most consistent predictors of later-life health outcome [6,8,12,14,20–31]. In particular, of these, childhood cognitive ability has been identified as the strongest determinant of later-life intelligence explaining ~50% of the variance in cognition even at age ~80 [9,25–27]. Consequently, a series of other possible determinants (physical activity, tobacco smoking, hypertension, obesity, reduced cardiac output, nutrition), with smaller, but significant effects on cognitive function and brain aging have been identified that may explain the remaining variability [6–11,17,18,22,32–35]. However, many of these measures have been branded as proxy markers of lower early-life intelligence.

For both diagnostic and research purposes, multimodal magnetic resonance imaging (MRI) is a popular choice for exploring age-related brain correlates of cognitive change [36–48]. To date, the resulting body of evidence converges on age-related decreases cross-sectionally and longitudinally, in the density of dopaminergic receptors [49], cortical thickness [44], whole-brain and regional volumes [46,50–52], and increases in ventricular volume [53], and the emergence of neural insults of cerebrovascular origin [33,36,54,55]. Furthermore, much of the age-related variation in brain structure has shown regional and temporal specificity, with frontal, parietal, and temporal lobes appearing most vulnerable to age-effects and the occipital lobe the least [8,10,56–62]. These findings are consistent with the anterior-to-posterior gradient of age-related brain deterioration – first coined in 1881 by French Philosopher Theodule Ribot when he introduced the concept of “Loi de regression” (i.e., last in, first out) – to describe memory formation and destruction [63]. Specifically, “first out” brain regions are characterized by a more complex architecture, a protracted ontogenetic developmental course, and are more likely to provide support when faced with neural insults, maladaptive brain function, or higher-order cognitive tasks [64–66].

Despite significant individual differences in aging trajectories, the overall consensus on age-related effects on brain health and cognition ability is clear: the brain shrinks with advancing age with alterations observed at both the molecular and morphological level, and these changes are linked to declines in specific cognitive domains [47,67,68]. Of these, processing speed, executive functioning, working memory, and inhibitory functions are the cognitive domains reported as most vulnerable to advancing age, whilst implicit procedural long-term memory, numerical processing, and the general knowledge accrued across the lifespan are those that appear relatively spared [6,7,9,10,37,47,68–70].

Currently, much of our knowledge on brain and behavior changes are derived from cross-sectional studies that compare single observations from individuals of different ages – most commonly groups of young and extremely old adults. Although suitable for identifying population-level mean trends, and efficient in terms of time and cost, cross-sectional studies are vulnerable to cohort effects, selection bias, and by design, can only offer insight on age-related-differences [37,43,50,52,62,65,71–73]. As aging research is essentially the study of change, a preferred approach of extracting individual differences in change – independently of individual differences in level – whilst simultaneously permitting the study of developmental and maturational trends is to use a longitudinal design with multiple follow-up assessments. Thus, to expand on prior efforts, we use within-subject (longitudinal) behavioral measurements that span across critical periods of the human lifespan using members of a prospective study; the 1953 Metropolitan Danish Male Birth Cohort (MDBC-1953) [14,74]. Specifically, following a major revitalization in 2009, research efforts based on MDBC-1953 data has focused on age-related cognitive decline. Here, the main aim is to elucidate why some older adult’s cognitive abilities are preserved well into late adulthood, while others demonstrate rapid decline.

In order to optimize the possibility of cognitive ability in a late-midlife being found to be associated with biological (or other) correlates, we exploited the long-term nature of this study to identify individuals with a relatively large decline in cognitive ability from early-adulthood (“decliners”), and those who show improvement (“improvers”). This standard approach, formally known as the Extreme Groups Design (EGD) [75], also maximizes the subject variability in other relevant factors such as education attainment, occupational complexity and levels of motivation, increasing the generalizability of this study to the real population. That is, in what is otherwise a highly homogenous cohort, the EGD attenuates the commonly observed selection bias of self-selected healthy study samples towards high-functioning and educated individuals. Further benefits of using this cohort are manifold. First, there is a lack of evidence suggesting that pathological change abruptly begins at old age after a period of relative stability. Thus, inclusion of childhood, youth and late midlife cognitive scores in aging studies may be key to predicting later-life health outcomes [8,13,67,68]. Second, a homogenous, late-midlife cohort provides conditions that are optimal for assessing the influence of potential candidate determinants on late-life morbidity without confounding cohort or other age factors. Third, findings from the extant literature exploring brain markers of developmental and aging processes have described age-associated changes as “early development in reverse” [42,64,66]. Thus, phenotyping across the human lifespan and not just the extremes of the age-range is optimal when exploring normative or pathological brain aging patterns [9,15,37,68,69,76]. Specifically, if the brain’s ‘blueprint for aging’ has already developed by preschool years, the conservative approach of selecting the oldest of old to expose biomarkers of normative aging is an outdated one [25–27]. Lastly, even when data are derived from a longitudinal study, many factors (demographic, lifestyle etc.) that may contribute to the observed heterogeneity in aging trajectories are ignored which ultimately undermines the reliability of the relationships discovered.

Considering this, our primary focus was to explore the factors—general health, vascular, demographic and lifestyle—that contribute to normative brain and cognitive aging. Crucially, we are not interested in just the age-dependence in any individual physiological or behavioral component, but a holistic range of endogenous and exogenous influences accrued across the lifespan. To achieve this, we distinguish between life course influences that act to preserve health (“positive influences”) versus those that are implicated in its demise (“negative influences”). This approach has the potential to identify specific brain and cognitive patterns that may underlie differential aging trajectories, whilst simultaneously exposing the relation of these patterns to a broad range of modifiable risk and protective factors. By modelling multiple variables of multiple modalities simultaneously we provide a more precise estimate of their synergetic effects filling a gap in the existing aging literature. Furthermore, our inclusion of both bivariate and multi-level analyses allows for both specific and general relations to be explored, which are potentially more informative than either approach alone. Specifically, this study goes beyond just investigating the interrelations among a selection of variables; rather, we are seeking specific age-related patterns of brain structure that are associated to sets of correlated cognitive, demographic, health, and behavior variables, as brain-behavior modes of population covariation.

## RESULTS

Participant characteristics are reported in Tables 1-4

**Table 1:** List and study sample characteristics of individual cognitive measures.

**TOTAL COGNITIVE MEASURES (N=31)**			
			
			
**INTELLIGENCE**		**GROUP A**	**GROUP B**
		***M* (SD)**	***M* (SD)**
	Härnquist (IQ-11)	78.0 (14.6)	70.4 (15.1)
	BP (IQ-20)	46.3 (9.6)	45.7 (8.2)
	IST2000-R (IQ-57)	42.7 (7.2)	21.3 (6.1)
	IST2000-R (IQ-63)	38.5 (7.9)	23.4 (7.6)
**COGNITVE DOMAIN**	**CANTAB**	**GROUP A**	**GROUP B**
		***M* (SD)**	***M* (SD)**
**Visual paired associates learning and memory**	**Paired Associates Learning (PAL)**		
	First trial memory score	18.2 (3.4)	16.5 (3.1)
	Total Errors Adjusted	17.8 (21.7)	23.3 (16.4)
	Total Trials Adjusted	13.2 (4.1)	14.8 (3.6)
**Pattern recognition memory**	**Pattern Recognition Memory (PRM)**		
	Percent correct	92.3 (8.0)	88.0 (8.6)
	Standard deviation correct latency (msec)	783.3 (477.3)	1036.1 (621.3)
**Spatial recognition memory**	**Spatial Recognition Memory (SRM)**		
	Percent correct	85.9 (8.4)	80.4 (8.9)
	Standard deviation correct latency (msec)	1357.0 (575.3)	1694.9 (977.9)
**Motor skills**	**Motor Screening (MOT)**		
	Mean Error	9.0 (2.1)	9.3 (2.2)
	Mean Latency (msec)	1129.0 (399.3)	1104.6 (274.0)
**Reaction time**	**Reaction Time (RTI)**		
	Mean 5-choice movement time	388.2 (88.1)	388.9 (107.4)
	Mean 5-choice reaction time	364.2 (47.1)	376.0 (53.3)
**Attention**	**Rapid Visual Processing (RVP)**		
	A' Score	0.9 (0.1)	0.9 (0.1)
	Mean latency block 1 (msec)	362.4 (88.6)	396.5 (140.6)
	Mean latency block 2 (msec)	332.7 (107.9)	358.2 (121.4)
	Mean latency block 3 (msec)	340.4 (68.6)	365.0 (124.7)
	Mean latency block 4 (msec)	414.4 (89.0)	444.2 (143.9)
**Global cognitive functioning**	**ACE**		
	Total Score	95.96 (3.2)	92.0 (5.0)
	**MMSE**		
	Total Score	29.5 (0.9)	29.2 (1.0)
**Executive function (planning)**	**Stockings of Cambridge (SOC)**		
	Problems solved in minimum moves	9.67 (1.5)	9.5 (1.4)
	Mean 5-moves	5.90 (1.0)	6.14 (1.0)
	Mean initial thinking time 5-moves (sec)	15.7 (10.2)	15.4 (16.1)
	Mean subsequent thinking time 5-moves (sec)	1.3 (2.3)	1.9 (2.7)
	**PAPER AND PENCIL TESTS**	**GROUP A**	**GROUP B**
		***M* (SD)**	***M* (SD)**
**Verbal paired associative learning and memory**	**15 Word Pairs Recall and Retention**		
	Learning	8.3 (7.7)	14.6 (8.2)
	Retention	3.5 (3.2)	6.0 (3.6)
**Processing speed**	Digit symbol modalities test	51.0 (7.4)	44.0 (8.4)
	Trail Making A (sec)	32.0 (10.7)	34.6 (9.6)
	Trail Making B (sec)	68.1 (20.8)	92.9 (61.5)
			

(Abbreviations: BP = Børge Priens Test; IST = Intelligenz-Struktur-Test 2000 R; CANTAB = Cambridge Neuropsychological Test Automated Battery; ACE = Addenbrooke’s Cognitive State Examination; MMSE = Mini Mental State Examination). Information on missing datasets include: Härnquist (IQ-11) = 39, BP (IQ-20) = 1, IST2000-R (IQ-57) = 1, IST2000-R (IQ-63) = 70, ACE = 1, Mean 5-choice movement time = 1, Mean 5-choice reaction time = 1, A' Score = 1, Mean latency block 1 (msec) = 1, Mean latency block 2 (msec) = 1, Mean latency block 3 (msec) = 1, Mean latency block 4 (msec) = 1. Total cognitive measures included n=31.

**Table 2:** List and study sample characteristics of social and biological demographic measures.

**TOTAL DEMOGRAPHIC MEASURES (N=8)**		
		
**Social**	**GROUP A**	**GROUP B**
	N (%)	N (%)
**Subject SEP**		
Working	50 (29.9%)	24 (12.4%)
Other	17 (8.8%)	17 (8.8%)
Early retirement	1 (0.5%)	4 (2.1%)
In education	1 (0.5%)	-
**Unknown**	**26 (13.5%)**	**53 (27.5%)**
**Paternal SEP**		
Self-employed, employee, or civil servant	51 (26.4%)	44 (22.8)
Skilled worker	14 (7.3%)	23 (11.9%)
Unskilled worker	24 (12.4%)	22 (11.4%)
**Unknown**	**6 (3.1%)**	**9 (4.7%)**
**Civil Status**		
Single (no)	61 (31.6%)	41 (21.2%)
Single (yes)	8 (4.1%)	4 (2.1%)
**Other/Unknown**	**26 (13.5%)**	**53 (27.5%)**
**Offspring**		
No	8 (4.1%)	4 (2.1)
Yes	61 (31.6%)	41 (21.2%)
**Other/Unknown**	**26 (13.5%)**	**53 (27.5%)**
**School Years/Education Attainment**		
Mean (SD)	11.4 (2.6)	10.2 (1.5)
**Unknown (%)**	**8 (4.1%)**	**14 (7.3%)**
**Biological**	**GROUP A**	**GROUP B**
	*M* (SD)	*M* (SD)
Birth length (cm)	51.8 (3.4)	52.8 (1.9)
Birth weight (g)	34.6 (5.2)	35.7 (3.9)
**Unknown**	**3 (1.6%)**	**5 (2.6%)**
		

(Abbreviations: SEP = social economic position; SD = standard deviation, g =grams). Total demographic measures included n=8.

**Table 3:** List and study sample characteristics of health measures.

**TOTAL HEALTH MEASURES (N=22)**			

**Prevalence of NCDs (self-reported)**		**GROUP A**	**GROUP B**
Asthma Cancer Cardiovascular Cerebrovascular Depression Diabetes Hypercholesterolemia Hypertension Migraine Prolapsed Disc		3 (1.6%)	2 (1.0%)
		1 (0.5%)	2 (1.0%)
		8 (4.1%)	9 (4.7%)
		4 (2.1%)	4 (2.1%)
		2 (1.0%)	4 (2.1%)
		2 (1.0%)	3 (1.6%)
		7 (3.6%)	7 (3.6%)
		22 (11.4%)	14 (7.3%)
		7 (3.6%)	2 (1.0%)
		4 (2.1%)	2 (1.0%)
**Prevalence of familial history of NCDs (self-reported)**			
Cardiovascular Cerebrovascular Dementia Diabetes Depression Hypertension Myocardial Infarct		14 (7.3%)	10 (5.2%)
		13 (7.3%)	10 (5.2%)
		17 (8.8%)	12 (6.2%)
		12 (6.2%)	8 (4.1%)
		18 (9.3%)	9 (4.7%)
		24 (12.4%)	10 (5.2%)
		14 (7.3%)	6 (3.1%)
**Common health biomarkers**		**GROUP A** ***M* (SD)**	**GROUP B** ***M* (SD)**
BMI (kg/m2) Height (cm) Weight (kg) Major Depression Inventory (MDI) score Cerebral Blood Flow (normalised to brain size) (mL/min) Total Cholesterol mmol/L		26.1 (3.3)	26.8 (3.2)
		180.5 (6.1)	179.8 (6.7)
		85.1 (12.0)	86.4 (11.6)
		4.0 (3.6)	4.9 (4.7)
		55.7 (13.1)	53.4 (13.9)
		5.5 (0.9)	5.6 (1.0)
			

(Abbreviations: NCDs = non-communicable diseases; BMI = body mass index; SD = standard deviation). Missing datasets: Prevalence of NCDs (self-reported) =12, Prevalence of familial history of NCDs (self-reported) = 12, BMI = 7, height = 7, weight = 7, MDI = 12, Cerebral blood flow = 12, Total Cholesterol = 8. Total health measures included n=22.

**Table 4:** List and study sample characteristics of lifestyle variables.

**TOTAL LIFESTYLE MEASURES (N=9)**		
	GROUP A	GROUP B
**Alcohol**	**N (%)**	**N (%)**
Status		
Yes	61 (31.6%)	39 (20.2%)
No	1 (0.5%)	2 (1.0%)
**Unknown**	**34 (17.4%)**	**56 (29.0%)**
	***M* (SD)**	***M* (SD)**
Start age	15.5 (1.7)	15.0 (1.9)
Units per week	11.8 (14.0)	9.2 (9.7)
**Unknown *N* (%)**	**34 (17.4%)**	**56 (29.0%)**
**Exercise (frequency)**	**N (%)**	**N (%)**
Daily	8 (4.1%)	17 (8.8%)
2-3 per week	29 (15.0%)	11 (5.7%)
1 per week	8 (4.1%)	4 (2.1%)
2-3 per month	3 (1.6%)	2 (1.0%)
Few per year	6 (3.1%)	1 (0.5%)
Never	6 (3.1%)	6 (3.1%)
**Unknown**	**35 (18.0%)**	**57 (29.5%)**
**Smoking**	**N (%)**	**N (%)**
Status (yes)	37 (19.2%)	28 (14.5%)
Status (no)	23 (11.9%)	12 (6.2%)
**Unknown**	**35 (18.0%)**	**58 (30.1%)**
	***M* (SD)**	***M* (SD)**
Smokes (pack/year)	17.3 (12.5)	18.2 (11.9)
Age start (years)	15.0 (2.4)	15.4 (4.4)
Age stop (years)	41.3 (11.6)	41.3 (13.1)
**Sleep quality**	***M* (SD)**	***M* (SD)**
Pittsburgh Sleep Quality Index	4.0 (2.0)	4.7 (2.7)
**Unknown**	**32**	**55**
		

(Abbreviations: SD = standard deviation.) Total lifestyle measures included n=9.

### Univariate analyses

### *Whole-group univariate associations*

Higher general cognitive ability in early adulthood (age 20) is significantly associated with greater grey matter (GM) volume in late midlife (age 57). Greater height is significantly associated with higher mode of anisotropy (MO) in the medial lemniscus. Higher general cognitive ability (assessed at ages 20 (IQ-20), 57 (IQ-57) and 63 (IQ-63), and a higher score in the Addenbrooke Cognitive Examination (ACE) are significantly associated with number of years in education.

**Figure 1:**
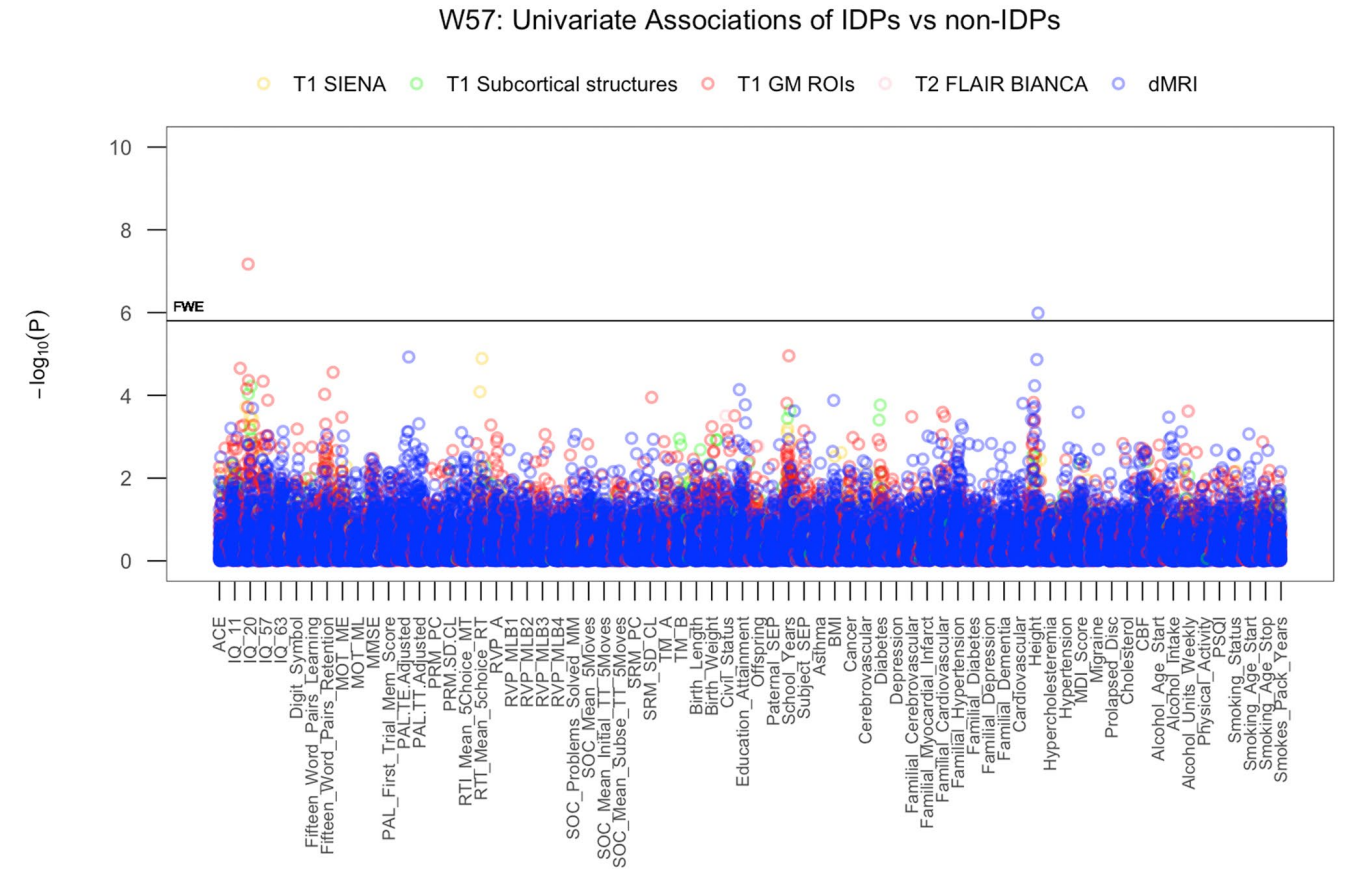
**The significance of associations between IDP and non-IDP variables.** The Manhattan plot shows all results for 453 IDPs against each of the 70 non-IDPs (31,710 values) adjusted for confounders: age, motion, and head size. Significance is plotted as -log _10_ p-values, arranged by non-IDPs on the x-axis, multiple testing thresholds across all pairwise associations are marked with a horizontal line (FWE: 5.8 x 10^-5^). IDPs are distinguished by plotting color to reflect the MRI modality and image processing tool used to estimate each measure. This created five IDP subdomains: 1) T1w global brain volume measures (normalized and unnormalized for head size) modelled by SIENAX (yellow), 2) T1w subcortical structures (shapes and volumes) modelled by FIRST (green), 3) T1w total grey matter volume within grey matter region-of-interests modelled by FAST (red), 4) T2w-FLAIR total volume of white matter hyperintensities modelled by BIANCA (pink), 5) dMRI estimates of diffusivity measures contained within 48 standard-space WM tract region-of-interests modelled by TBSS (blue). (Abbreviations: IQ-11, IQ-20, IQ-57, IQ-63 = general intelligence scores at ages 11, 20, 57, and 63; MOT = motor task; ME = mean error; ML = mean latency; PAL = paired associates learning; TE adjusted = total errors adjusted; TT Adjusted = total trials adjusted; PRM = pattern recognition memory; SD = standard deviation; CL = correct latency; RTI = reaction time task; MT = movement time; RT = reaction time; RVP = rapid visual processing task; MLB1-4 = mean latency block 1 to 4; SOC = Stockings of Cambridge task; Mean Initial TT 5 Moves = mean initial total time 5 moves task; Mean Subse TT 5 Moves = mean subsequent thinking time 5 moves task; SRM = spatial recognition memory; TM = trail making task; SEP = social economic position; MDI = Major Depression Inventory; CBF = cerebral blood flow; PSQI = Pittsburgh Sleep Quality Index.

We visualize results with Manhattan plots that show -log_10_ p-values for IDP-by-non-IDP correlations, arranged by non-IDPs on the x-axis, multiple testing thresholds across all pairwise associations are marked with a horizontal line (here only familywise error rate (FWE) is indicated as False Discovery Rate (FDR) identified no additional significant result). We identified two significant univariate associations between specific imaging derived phenotypes (IDP) and non-imaging derived phenotypes (non-IDP) both non-adjusted and adjusted for the effects of cognitive change, Figure1 and Supplementary Figure 7A-7B. Specifically, we found that higher scores in early adulthood IQ (IQ-20) is associated with a greater GM volume in the right temporal pole. Additionally, we identified a positive association between height and MO in the medial lemniscus (left). Table 5 lists all FWE-significant correlations extracted from Figure 1, (FWE threshold: p-uncorrected = 5.80 x 10^-5^).

**Table 5:** List of significant univariate associations.

**A**			
**IDP**	**NON-IDP**	***r***	**p-uncorrected**
			
**T1 GM ROI**	**Early Adulthood IQ (BP Test)**		
Volume of GM in Temporal Pole (R)	IQ-20	0.38	6.73E-08
**dMRI**	**Health**	0.49	1.03E-06
MO in Medial Lemniscus (L)	Height (cm)		
			
**B**			
COGNITIVE MEASURE	ALL (other) NON-IDPs	r	p-uncorrected
			
**Early Adulthood IQ (BP Test)**	**Education**		
IQ-20	No of years	0.53	1.98E-08
**Late Midlife IQ (IST)**	**Education**		
IQ-57	No of years	0.49	3.57E-07
IQ-63	No of years	0.44	4.18E-06
**Global Cognitive Functioning**	**Education**		
ACE	No of years	0.43	1.69E-05

**Figure 2:**
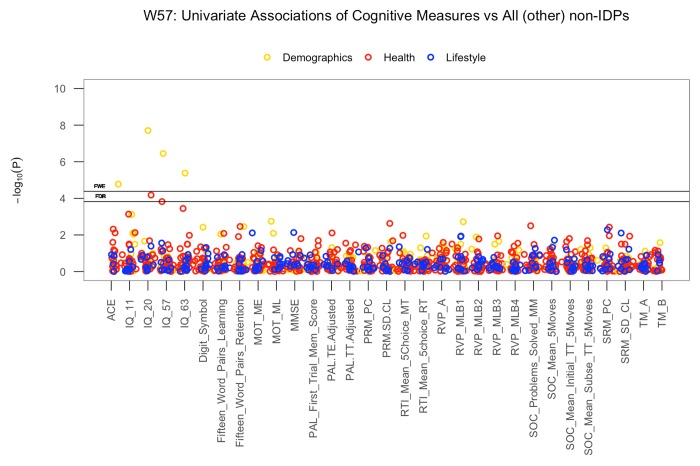
**The significance of associations between each cognitive measure and all (other) non-IDP variables.** The Manhattan plot shows all results for 31 cognitive variables against each of the 39 (other) non-IDPs (1209 values) adjusted for confounders: age, motion, and head size. Significance is plotted as -log _10_ p-values, arranged by cognitive variables on the x-axis, multiple testing thresholds across all pairwise associations are marked with a horizontal line, FWE top line and FDR bottom line (FWE threshold: 4.38 x 10^-4^; FDR threshold: 3.82 x 10^-3^). All other non-IDPs are distinguished by plotting color (demographic = yellow, health = red, lifestyle = blue). (Abbreviations: IQ-11, IQ-20, IQ-57, IQ-63 = general intelligence scores at ages 11, 20, 57, and 63; MOT = motor task; ME = mean error; ML = mean latency; PAL = paired associates learning; TE adjusted = total errors adjusted; TT Adjusted = total trials adjusted; PRM = pattern recognition memory; SD = standard deviation; CL = correct latency; RTI = reaction time task; MT = movement time; RT = reaction time; RVP = rapid visual processing task; MLB1-4 = mean latency block 1 to 4; SOC = Stockings of Cambridge task; Mean Initial TT 5 Moves = mean initial total time 5 moves task; Mean Subse TT 5 Moves = mean subsequent thinking time 5 moves task; SRM = spatial recognition memory; TM = trail making task).

List of IDP-by-non-IDP (A) and cognition-by-all (other)-non-IDP (B) associations passing Bonferroni correction threshold extracted from Figure 1 (5% FWE: p-uncorrected < 5.80 x 10^-5^) and Figure 2 (5% FWE: p-uncorrected < 4.38 x 10^-4^) respectively. (Abbreviations: Non-IDP = non-image derived phenotype, IDP = image derived phenotype, BP Test = Børge Priens Test, IST = Intelligenz-Struktur Test 2000 R, GM = grey matter, ROI = region-of-interest, dMRI = diffusion Magnetic Resonance Imaging, R = right, L = left).

### *Validation test: subgroup univariate associations*

Results exploring univariate associations for each sub-group separately (i.e., subgroup group A improvers, and subgroup group B decliners) indicated that the significant associations observed at the level of the whole-group where largely driven by measures derived from subgroup A members. This suggests that, despite both groups being relatively homogenous, it is variation in the improvers that are most responsible for the observed effects. Crucially, we did not observe inconsistent effects in the sign of effects that would suggest Simpsons Paradox. Having evidence that the EGD is not inducing paradoxical effects, we can further explore IDP-by-non-IDP relations controlling for changes in IQ.

### *Univariate associations: adjusting for cognitive change (C∆)*

Univariate associations estimating the relation between each of the IDP and non-IDP measures after adjusting for the effects of C∆1 (IQ-57-IQ-20) revealed negligible deviation from the whole-group results visualized in Figure 1. However, when adjusting all variables for the effects of C∆2 (IQ-63-IQ-57), we observed modest attenuation of the IQ-20 and GM volume of the temporal pole (right) association, Supplementary Figure 7A, but negligible change to the association between height and MO in the medial lemniscus. Conversely, we found that adjusting for C∆3 (IQ-20-IQ-11) did not affect the significant link between IQ-20 and GM volume of the temporal pole, but did remove the significant correlation between height and MO in the medial lemniscus. Similarly, when estimating the relationship between each cognitive variable and all (other) non-IDP measures after adjusting for the effects of C∆1 and C∆2 we found negligible changes to the associations visualized in Figure 2. However, adjusting for the effects of C∆3 resulted in the removal of all prior significant associations listed in Table 5.

### Multivariate associations

### *Whole-group multivariate associations*

Results from CCA identified a single statistically significant mode of population co-variation coupling individual cross-subject variations in brain structure to an extensive range of non-imaging measures (R_c_ = 0.75, permuted p-corrected = 0.01). Post-hoc analyses revealed IQ variables, SEP, psychosocial factors and GM volume as most influential in driving the multivariate correspondence between IDPs and non-IDPs.

For ease of interpretation, we invert the signs of all non-IDP measures where lower outcome values are indicative of a positive quality or indicator (e.g., cognitive tests measuring reaction time or number of errors, number of smokes etc.). Thus, when interpreting post-hoc correlations between each non-IDP and the CCA-mode, Figure 3A, all positive correlations describe positive contributions to the CCA-mode (e.g., higher cognitive ability, higher paternal SEP, better health and lifestyle choices), whilst all negative correlations identify unfavorable contributions to the CCA-mode. In view of this, we report the strongest positively associated non-IDP variable to the CCA-mode as IQ-20 (*r^2^* = 18.8%, *r*=0.43) and the strongest negatively linked variable as the motor task (time taken) (*r^2^* =6.9%, *r* =-0.26). Other strong positive non-IDP contributions to the CCA-mode include cognitive variables assessing verbal paired associative learning and memory (15 word pairs learning and retention), attention with working memory load (RVP), measures of general IQ from childhood (IQ-11) and late midlife (IQ-57 and IQ-63), global cognitive functioning (ACE, MMSE), executive function and planning (SOC), motor and reaction time (RTI), and health and sociodemographic variables (major depression inventory (MDI) core, self-reported history of depression, and paternal SEP). Conversely, we identified an appreciably smaller number of strong negative non-IDP contributions influencing the underlying structure of the CCA-mode. Of these, civil status (where a ‘non-single’ status was coded as a positive variable) and smokes (packs per year), were found to be most influential.

**Figure 3A:**
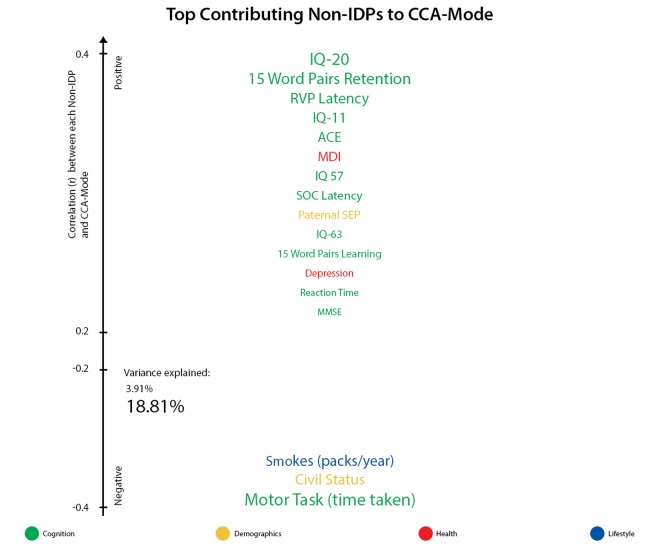
**Top contributing non-IDP variables to CCA-mode.** Individual non-IDP measures most strongly associated with the identified CCA-mode of population covariation. The CCA-derived weights visualized indicate how much each measured variable contributes to the significant CCA-mode i.e., the measure of the strength of involvement of an observed variable to the CCA-mode. Non-IDPs are colored according to their assigned subdomains (cognition = green, demographic = yellow, health = red, lifestyle = blue). The vertical position of each variable is related to the scale of the association of that specific measure with the identified CCA-mode. Font size is indicative of variance explained by the CCA-mode. Here we do not report variables that attain a correlation value between 0.2 to -0.2.

With regards to post-hoc correlations computed between IDPs and the CCA-mode, Figure 3B, we identified GM volume of the temporal pole (left and right) as the strongest positively linked brain-imaging marker (*r^2^* = 29.3%; *r* =0.54), and axial diffusivity L1 of the posterior limb (right) of the internal capsule as the strongest negatively linked (*r^2^* = 7.45%; *r^2^* =-0.35). Further top positive IDP contributions were succeeded by global brain volume measures of GM, white matter (WM) and peripheral cortical GM, a broad range of other GM region-of-interest (ROI) volume measures, and volume of the sub-cortical structure, the thalamus. Similar to non-IDPs, strong negatively contributing IDPs were fewer in number, such that the identified CCA-mode largely related positively contributing brain macrostructural markers (i.e., measuring larger whole-brain GM and WM volume, subcortical structure volumes, or GM volume of ROI) to each other and to range of diffusion MRI (dMRI) tract-based spatial statistics (TBSS) derived microstructural markers.

**Figure 3B:**
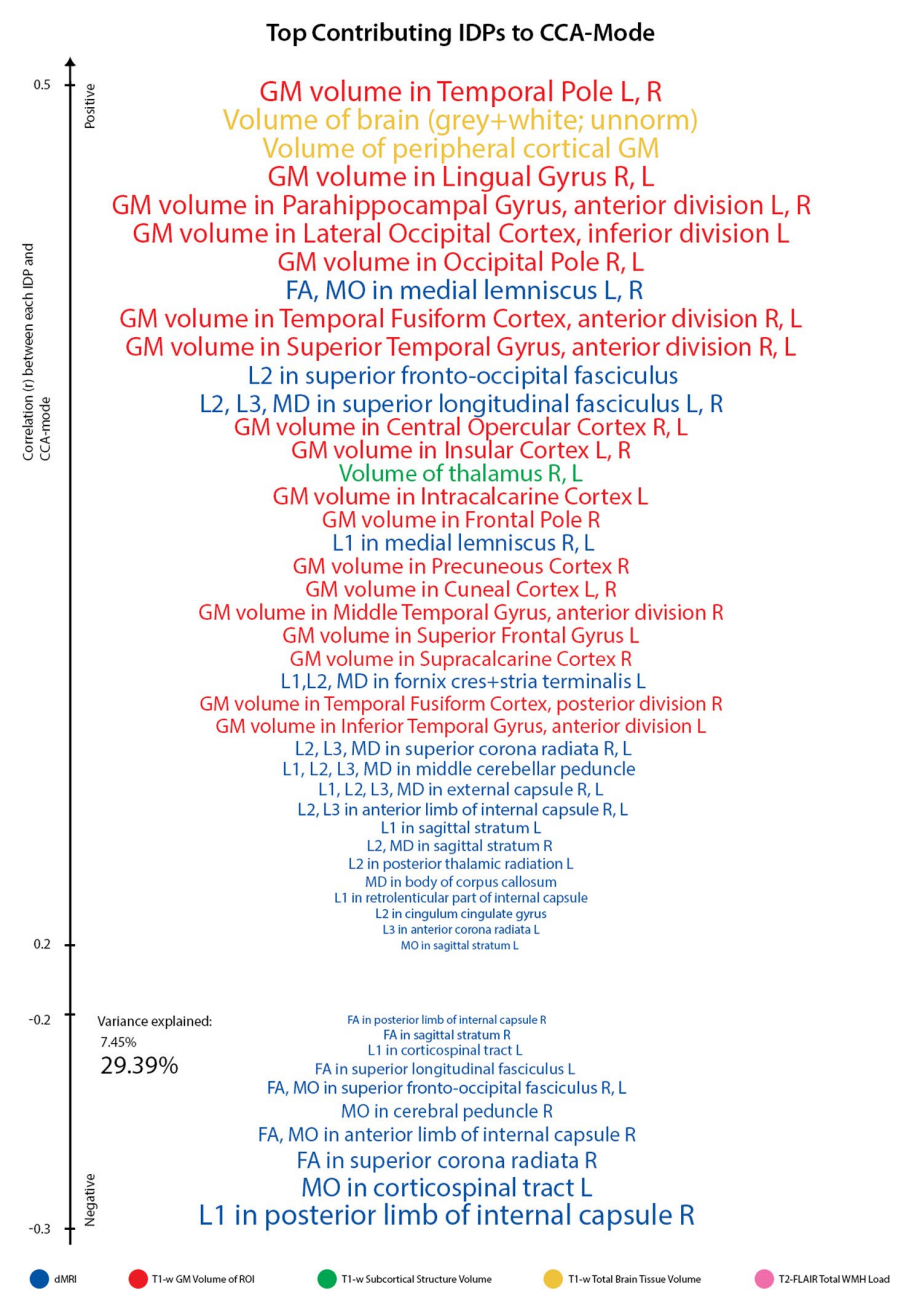
**Top contributing IDP variables to CCA-mode.** Individual IDP measures most strongly associated with the CCA-mode of population covariation. The CCA-derived weights visualized indicate how much each measured variable contributes to the significant CCA-mode i.e., the measure of the strength of involvement of an observed variable to the CCA-mode. IDPs are colored according to their assigned subdomains: dMRI-TBSS = blue, T1w-FAST total grey matter volume within grey matter region-of-interests = red, T1w-FIRST subcortical structure volumes = green, T1w-SIENAX total brain tissue volume = yellow, T2w-FLAIR-BIANCA total volume of white matter hyperintensities = pink. The vertical position of each variable is related to the scale of the association of that specific measure with the identified CCA-mode. Font size is indicative of variance explained by the CCA-mode. Here we do not report variables that attain a correlation value between 0.2 to -0.2. (Abbreviations: L = left, R = right, FA = fractional anisotropy, L1 = 1^st^ eigenvalue, L2 = 2^nd^ eigenvalue, L3, = 3^rd^ eigenvalue, MD = mean diffusivity, MO = tensor mode).

Figures 4A and 4B visualize the importance of each subdomain in influencing the multivariate associations underpinning the significant CCA-mode. In brief, for each subdomain (x-axis), we compute the mean observed-variable-to-CCA-mode correlation across all variables in that subdomain. This value is plotted on the y-axis using units of correlation (*r^2^*). Specifically, the length of each bar represents that subdomain’s average importance – both positive (grey) and negative (black) – to the identified CCA-mode. Similar to the interpretation of post-hoc correlations in Figure 3A, in Figures 4A and 4B we invert the signs of both non-IDP and IDP measures where a lower value is indicative of a “positive” trait/marker and a higher value is representative of a “negative” trait/marker, such as in the case of DTI-derived indices MD, L1, L2, and L3, or cognitive tasks measuring reaction time or number of errors. Thus, positive correlations between a given subdomain and the CCA-mode represents categorially-driven contributions from “positive” IDP and non-IDP markers, whilst negative correlations represent categorically-driven contributions pertaining to “negative” traits. With this in mind, the subdomains dominating the underlying CCA-based associations were identified as positive contributions from total brain tissue volume (*r^2^*=4.41%), GM volume of non-cerebellum ROIs (*r^2^*=3.16%), subcortical structure volumes (*r^2^*=2.56%), and cognition (*r^2^*=3.24%). With regards to meaningful links between subdomains, our results identified a mode of population covariation that resembles the general intelligence g-factor (i.e., the observed commonality among observed mental abilities [77]) but which also includes a broad range of other non-cognitive variables describing traits related to biophysical, sociodemographic, lifestyle and health factors. In addition, the identified CCA mode largely distinguishes between subject performance in the various measures included, allowing their relative contribution to the CCA-mode to be described as either positive or negative. In this regard, the identified mode can be represented by a “positive-negative” axis that links positive measures of cognitive performance to each other and to a meaningful pattern of other (non-imaging) measures (e.g., better performance in cognitive tests, higher educational attainment, regular physical activity, higher SEP vs measures of lower cognitive performance, lower education attainment, physical inactivity and poorer health status etc.).

**Figure 4A:**
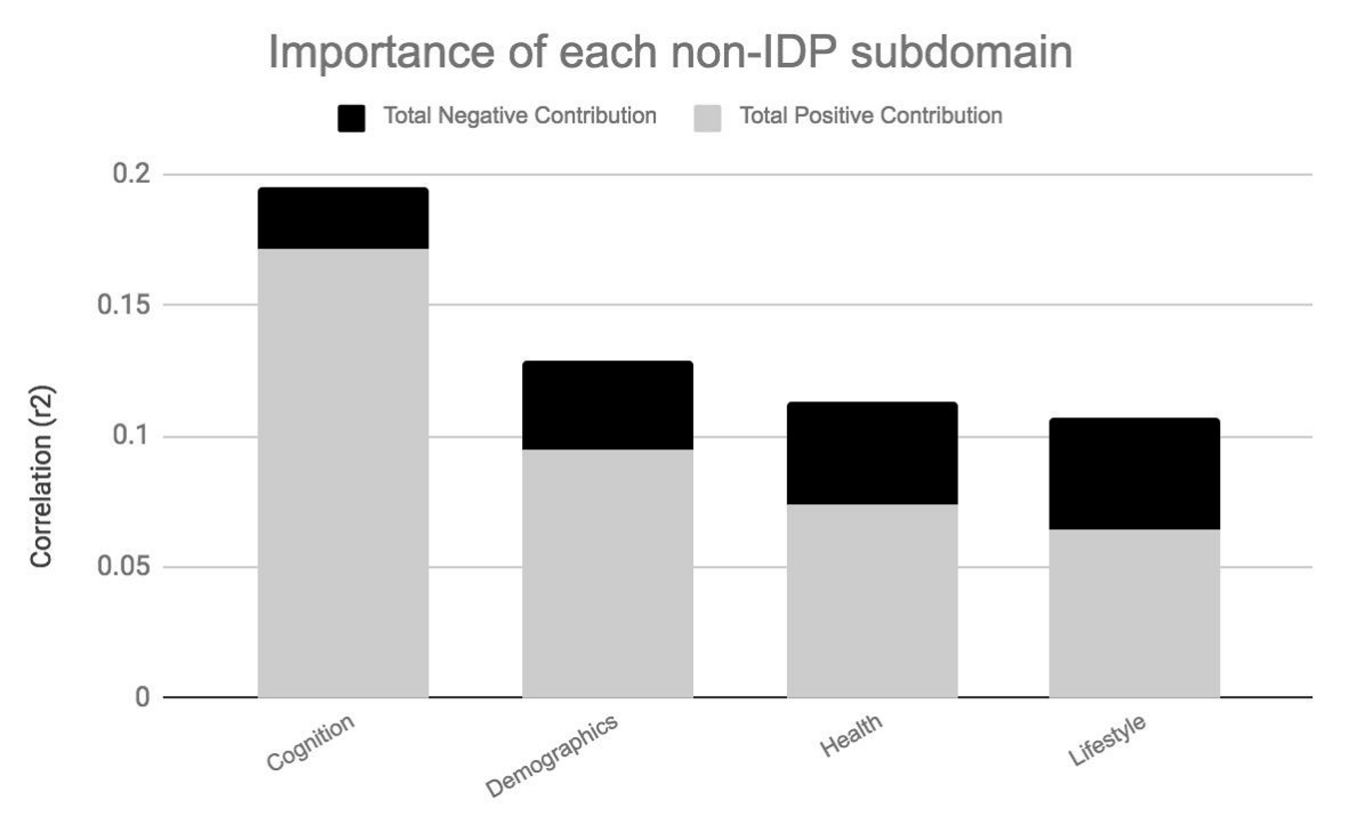
**Importance of non-IDP subdomains to CCA-mode.** Figure 4A visualizes the overall significance of non-IDP subdomains in influencing multivariate associations between each variable included in the measurement battery. For each subdomain (x-axis), the length of each bar represents the average subdomain importance (r^2^) to the CCA-mode. Categorically-driven contributions from positive qualities or indicators are represented in grey, whilst contributions from negative traits are depicted in black. In this study, individual non-IDP measures derived from the subdomain cognition (3.24%) were the most important contributors to the CCA-mode of population covariation identified. (Abbreviations: Non-IDP = non-imaging derived phenotypes).

**Figure 4B:**
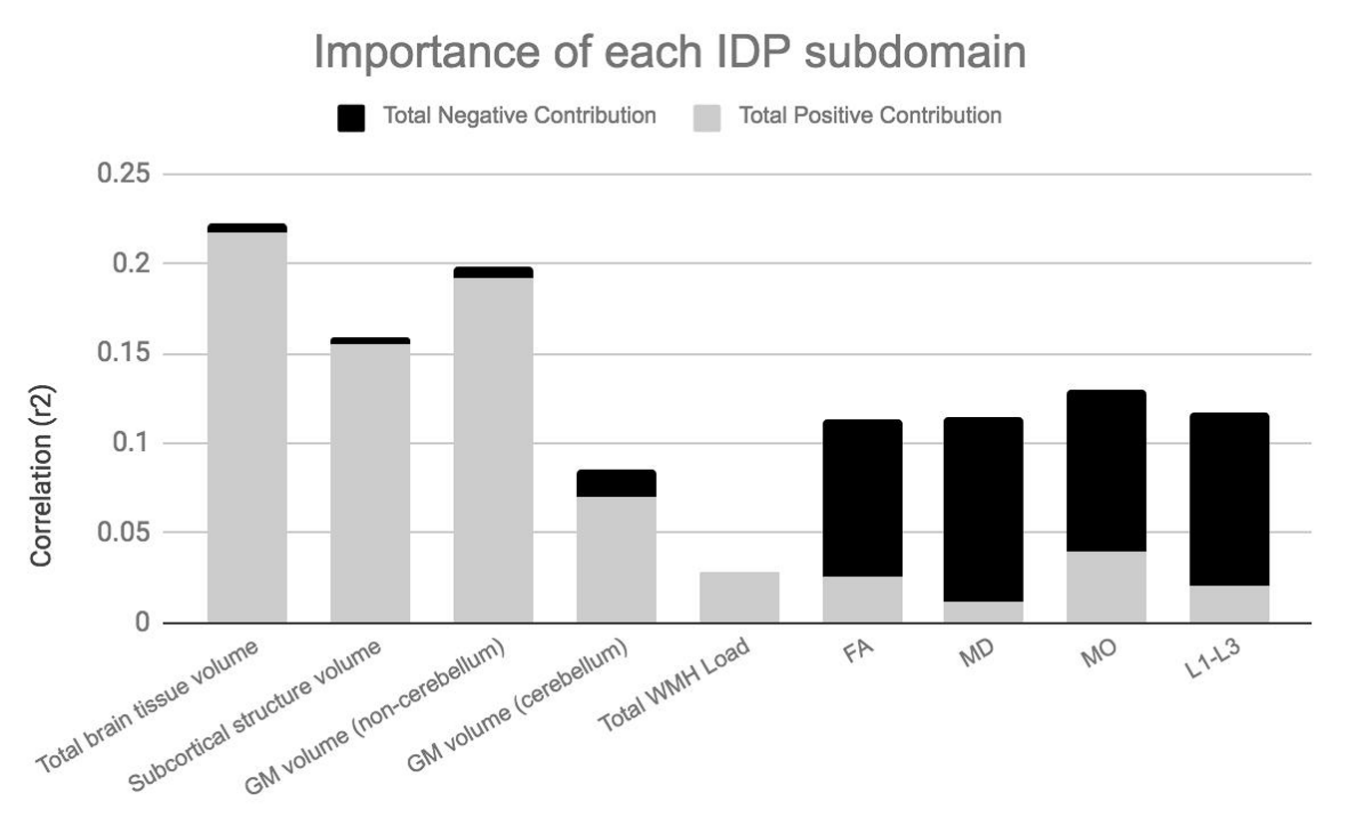
**Importance of IDP subdomains to CCA-mode.** Figure 4B visualizes the overall significance of IDP subdomains in influencing multivariate associations between each variable included in the measurement battery. For each subdomain (x-axis), the length of each bar represents the average subdomain importance (r^2^) to the CCA-mode. Categorically-driven contributions from positive qualities or indicators are represented in grey, whilst contributions from negative traits are depicted in black. In this study, individual measures derived from IDP subdomains total whole brain tissue volume (4.41%), GM volume of non-cerebellum ROIs (3.61%) and subcortical structure volumes (2.56%) were the most important contributors to the CCA-mode of population covariation identified. (Abbreviations: IDP = imaging derived phenotypes, GM = grey matter, WMH = white matter hyperintensity, FA = fractional anisotropy, L1 = 1^st^ eigenvalue, L2 = 2^nd^ eigenvalue, L3, = 3^rd^ eigenvalue, MD = mean diffusivity, MO = tensor mode).

We explored the multivariate results in a number of ways to establish that the estimated CCA-mode was not unduly influenced by the EGD. First, a scatterplot of the IDP and non-IDP canonical variates, with group membership indicated by plotting symbol, showed no evidence of clustering (Supplementary Figure 9). Next, we computed post-hoc correlations for each subgroup separately. Here the subgroup analysis identified similarities in the top contributing IDP and non-IDP measures to the significant CCA-mode, Supplementary Table 2. Supplementary Figure 10 also presents a stratified version of the variance explained plots shown in Figure 4A, Figure 4B again showing a generally similar pattern of contribution of each subdomain in the total, and two subgroups. Lastly, in order to further assess the CCA similarity in the subgroup analysis, we also provide a coefficient of factor congruence between groups for IDPs and non-IDPs. Here we identified a congruence value of 0.71 for non-IDPs and 0.75 for IDPs between groups.

Table 6 presents the overlap between bivariate and multi-levels findings.

**Table 6:** Overlapping significant univariate and multivariate associations.

	**UNIVARIATE ANALYSIS (*r*)**	**MULTIVARIATE ANALYSIS (*r*)**
NON-IDPs	IDP	CCA-Mode
		
**Intelligence Test (BP Test)**	Volume of GM in Temporal Pole	CCA subject weights
**IQ-20**	0.380	0.434
		

List of corresponding significant associations identified by univariate methods (column 2), and post-hoc associations between the significant CCA-mode of population covariation and individual (observed) variables (column 3). (Abbreviations: BP Test= Børge Priens Test, GM = grey matter).

## DISCUSSION

This study identified significant associations that linked multiple measures of brain neurostructure to an extensive battery of behavioral measures derived from the MDBC-1953 dataset. Specifically, this battery includes measures from several cognition domains, demographic, health and lifestyle factors. The results indicate that variance is shared across many aspects of behavior, and that this covariation may be of importance to the variability observed in brain structure. Fundamentally, this finding offers a better understanding of the types of variability that may exist across healthy aging individuals, as well as the factors that may underlie the observed differences. Specifically, using post-hoc correlations, we mapped the original behavioral and brain-imaging measures onto the significant CCA-mode of population covariation. Here, we provide a measure of importance of each observed variable in maximizing the relation between multiple brain structural and behavioral variables. The results indicated that the CCA-based associations were driven largely by cognitive ability, early-life SEP, psychosocial factors, and whole brain (GM and WM) tissue volume. The ability to identify individual indicators that may be of significance to the variability observed in healthy aging trajectories is extremely valuable to age-related biomarker research and warrants further investigation.

Many aging studies apply statistical models that are unable to consider the cumulative effect of various lifespan experiences on an outcome variable of interest. As age-related change is a continuous process, studies focusing on specific candidate determinants (e.g. GM volume, BMI, smoking status, amyloid load) limit the possibility of discovering new and unforeseen relations. To avoid such partial interpretations and expand on what is currently known about differential aging trajectories, we employed CCA, a multivariate technique to seek patterns of covariation between two sets of measures – i.e. IDPs and non-IDPs - simultaneously. A strength of this approach is that it boosts power by implicating the full dataset to investigate our main aim - the relation of brain structural markers with a set of behavioral measures and to further evaluate these relations with respect to a broad range of modifiable risk and protective factors. Analogous to the positive-negative mode previously identified in the HCP data [78,79], we identified a single significant mode of population covariation largely linking multiple brain imaging markers to (other) behavioral measures (*r*= 0.75, p-corrected = 0.01). Specifically, CCA coupled higher scores in general intelligence (IQ-20 and IQ-11), attention with working memory load (RVP), explicit verbal memory (15-Word Pairs Retention), global cognitive functioning (MMSE, ACE), executive functioning (SOC), reaction time (RTI), psychosocial well-being (MDI), self-reported prevalence of depression, and paternal SEP with larger total brain volume (GM and WM), larger volume of subcortical brain structures, and a range of WM microstructural brain indices.

To our knowledge, this work is the first to underscore psychosocial well-being and early-life conditions as significant contributors to the strength of brain and behavior associations observed in late-midlife. However, the discovery that individual variations in early-life SEP and psychosocial health may be most predictive of brain health and cognitive ability in later-life is consistent with the concept of a socioeconomic gradient in health which reflects how the most disadvantaged groups are also those at increased risk of disease [80,81]. Previous studies also highlighting the significance of social inequality in disease progression and premature mortality have attributed their observations to unknown influences termed “psychosocial factors” (i.e. hostility, depression, hopelessness), that act as mediators of SEP effects on later-life heath outcome [12,23,82–86]. Furthermore, it has been suggested that many of these overlooked measures may act independently of common vascular risk factors (VRFs) and non-communicable diseases but are still somehow ‘entangled’ within one’s SEP [82]. Finally, although smoking is a well-established risk factor for brain and cognitive decline [17,23,33,87], this was not observed in our study sample. The lack of significant effects may be explained by our moderately sized sample, errors of measurement, or the cerebral and/or cognitive benefits of giving up smoking during adulthood [6]. Notably, as we do not quantify the number of previous smokers in this study, we remain cautious about inferring on the health benefits of smoking cessation on later-life health outcome.

This study also identified top contributions from brain structural measures (i.e. brain biomarkers specifically important in maximizing the CCA-based associations) to cognitive performance that are in agreement with previous reports [8,10,37,38,56,60,64,67,76]. Specifically, we identified spatially diverse brain influences pertaining to temporal, frontal, occipital, forebrain volumes to variations in specific cognitive domains, verbal learning and memory, attention, reaction time, executive function, and global cognitive performance. Specifically, the strong contributions observed from the fronto-temporal cortices are in line with the idea of compensatory neural mechanisms that are recruited to provide additional support in response to age-related WM deterioration [10,13,15,60,67,88]. As many cognitive abilities evidence of age-effects by early to late midlife [68], it is plausible that the positive total brain volume contributions reflect neurostructural strategies that seek to maintain homeostatic cognitive function in the face of age-related cognitive decline. This finding is also consistent with several other conceptual models of cognitive aging [69,88,89]. However, although, these models predominantly describe functional recruitment in response to age-related neural insults, continuous task demands and functional deterioration, it has also been expanded to include structural changes and neurogenesis (e.g. in the study of structural changes in the hippocampi of London taxicab drivers [90]). Specifically, in this present study we identified a pattern of WM microstructure that was characterized by decreased directional coherence (i.e., decreased FA and MO), and increased mean diffusivity (increased MD, λ_ax_, and λ_rad_) in WM tracts that typically demonstrate increased vulnerability to age-effects (e.g. superior fronto-occipital fasciculus (SFOF), the superior corona radiata, superior longitudinal fasciculus, body of the corpus callosum, crus fornicis and stria terminalis (FC/ST), and sagittal stratum [40,91,92]. Guided by prior research reporting histological-DTI relations, the WM diffusivity patterns observed are consistent with the “last in, first out” hypothesis and may reflect age-related neurobiological processes such as chronic WM degeneration, demyelination, secondary Wallerian degeneration, or gliosis [25].

In age- or disease-related neurodegeneration, increases in MD (a sensitive but unspecific marker of cellular degeneration) are commonly linked to reduced FA and MO (reflecting an overall decrease in WM tract organization). However, we note that our results did not always identify such patterns of relations. In fact, previous studies have suggested that the relationship between DTI-derived summary measures may be region-dependent, and may not, especially in the case of older adults or patient groups, uniformly link decreases in anisotropic diffusion to increases in mean diffusion [92]. Furthermore, we found that the component measures, λ_rad_ and λ_ax_, demonstrated more extensive relations to non-IDP measures than their derivatives (FA, MO and MD). This finding has also been highlighted in previous studies where MD and other directional diffusivities, but not FA, presented strong relations to cognitive function [91]. In general, we found that the inclusion of component diffusivity measures serve as useful additional sources of information regarding WM integrity. However, we note that in regions of crossing fibers the interpretation of diffusion MRI measures should be made with respect to the underlying WM anatomy and the differential neuropathology of WM tracts [93]. The importance of this was demonstrated in an earlier imaging-study comparing changes in DTI-derived summary measures in areas of crossing fibres pertaining to data from Alzheimer disease (AD), MCI, and healthy aging subjects [93]. Specifically, this group discovered atypical increases in FA and MO in the MCI group compared to the healthy aging controls. Upon further examination using probabilistic tractography, they attributed the unusual rise in mode and fractional anisotropy to a region of crossing fibres in the centrum semiovale. Here, they found that the relative sparing of motor-related projection fibres that traverse the age-sensitive cognitive-related association fibres of the superior longitudinal fasciculus (SLF) artificially inflated FA and MO in the direction of the newly assigned primary eigenvector. Specifically, this finding provides a plausible explanation for the unexpected increases in FA and MO in conditions where the opposite is expected (i.e. decreases in anisotropic diffusion in disease conditions), underscoring the importance of interpreting DTI measures with respect to the underling WM microstructure. Consistent with the findings of Douaud et al, we also discovered linked increases in FA and MO in brain regions where mean diffusivity measures were also increased. Considering the late-midlife age-range of participants – one where negative age-related effects on cognitive ability and brain structure are commonly observed [68] – it is most likely that our findings also evidence very early pathogenic alterations of WM in complex brain regions such as crossing fibres. Similarly, we note that findings of increased FA and MO did not always extend to coupled increases in axial (λ_ax_ = λ1) and radial (λ_rad_ = λ2, λ3) diffusivity as would be expected in a healthy study samples. However, as the interpretation of diffusion anisotropic indices – FA and MO – in regions of crossing fibres are extremely complicated, it is likely that the interpretation of eigenvalues is equally convoluted and warrants further investigation.

Other factors that may explain the relations between variables that were in the opposite direction to what one would expect includes an insufficient lag time between changes in a brain biomarker, and their presumed effect on another variable(s). That is, the rate of effect of WM changes on a hypothesized effect variable (e.g., cognitive function) may not be instantaneous. In this case, future investigations that employ lead-lag analyses - using more than two assessments - are necessary to ensure that the observation interval harmonizes with the timing of the critical events. Alternatively, similar to findings from animal aging models [2], we also consider the possibility that the observed increases in regional brain volume may instead reflect pathological processes such as gliosis or defective elimination of by-products [76], and not compensatory neural mechanisms. In this regard, the link between positively contributing total brain volume measures and predominately negatively contributing dMRI indices would be largely explained. Lastly, previous studies have shown that the GM and WM contrast immediately below and above the WM surface is markedly reduced in healthy aging participants [94]. It is thus a possibility that methodological procedures concerning segmentation of brain tissue could have led to an overestimation of GM content and underestimation of WM content further accounting for the anti-correlation observed between the two tissues.

Univariate IDP-by-non-IDP associations that survived correction for multiple testing were few; however, those identified were in agreement with previous studies that also linked better cognitive ability to dispersed brain patterns of regional influence [6,9,10,17,57,62,76,95–98]. Specifically, we found a positive association between GM volume of the temporal pole and performance in a test of intelligence measured at age ~20 years (IQ-20). We also report a significant positive association between MO in the medial lemniscus and height. However, although previous studies have similarly identified a positive link between height and intact WM integrity [99], we also consider alternative interpretations that may have produced similar findings. Namely, as described in the case of [93], the observed co-localized increases in MO and FA in the medial lemniscus may indicate evidence of selective degeneration of secondary WM tracts which result in the “spared” fibers of this sub-region establishing a newly acclaimed primary eigenvector, and with it a misleading increase in both FA and MO [40].

We also identified few pairwise cognition-vs-all-(other)-non-IDPs associations that survived thresholds for multiple testing. Of these, the results confirm the well-acknowledged positive relations between cognitive ability (measured by IQ tests at ages 20, 57 and 63 and Addenbrookes Cognitive Examination at age 57) and educational achievement [100]. Interestingly, the results also revealed a strong positive link between IQ and height. Previous studies that have similarly identified links between intelligence and height have indicated that height may be a useful proxy marker for adverse early-life conditions and increased later-life dementia risk [101,102]. Although notably, the effect of genetic factors on height, IQ, and early life environmental conditions should also be considered. Finally, the results of validation tests regarding the effect of EGD on univariate associations showed the significant relations observed at the whole-group level were not driven by group average differences (and hence not by the extreme-group-design either). However, we found the whole-group associations were mainly representative of group A members – the “improvers” and not group B members – the “decliners”. Although a similar trend of relations does exist for group B members we speculate that at present the effect sizes do not meet the threshold for ‘discovery’.

Next, we explored univariate IDP-by-non-IDP associations after adjusting for change in IQ pertaining to both early-life and late-midlife. We found that IDP-non-IDP associations were largely unchanged when adjusting for the effect of C∆1 (IQ-57-IQ-20). However, adjusting for the effects of C∆2 (IQ-63-IQ-57) and C∆3 (IQ-20-IQ-11) revealed modest changes, Supplementary Figure 7A-7B. As early life intelligence is reputed to – in part – drive many of the observed relations between a “variable X versus cognitive performance” – an example of reverse causation [9,103,104] – we anticipated that adjusting for the effects of C∆3 may have attenuated or removed the significant relationship between IQ-20 and GM volume of the temporal pole. However, this was not observed, and the IQ-20-temporal-pole association remained significant. Conversely, we found that adjusting for the effects of C∆3 removed the association between height and MO in the medial lemniscus suggesting collinearity between C∆3 and one or more of the other measures in the model. As the association between height, brain and intelligence is largely attributed to shared genetic influences [105], the present findings should not be directly interpreted as evidence for change in early-life intelligence or early-life conditions affecting the height-brain relationship, but perhaps the effect of genetic factors that are interacting with change in cognitive function and/or environmental influences. Similarly, adjusting cognition-by-all-non-IDP correlations, Figure 2, for the effects of C∆1 and C∆2 resulted in negligible changes. However, accounting for the effects of C∆3 removed all prior significant correlations, Supplementary Figure 7C. Here, our results indicate that early-life cognitive change may play a role in the positive association identified between general cognitive ability and education. However, similar to the significant IDP-non-IDP associations, there are numerous likely causes of the IQ and education achievement association which principally concern variation in genetic profiles, and shared and non-shared environmental factors - with the former influential in the link between IQ and education attainment, and the latter important in the differences between them [106].

Notably, in this study we implemented two distinct methods for calculating cognitive change: 1. “raw difference scores” (RDS) formed by subtracting post-test scores from pre-test scores and 2. “residualized change approach” (RCA) which differences the observed score at follow-up (i.e., IQ-57) from the predicted score X_2_ (e.g., X_2_ is predicted with a linear regression analysis of the follow-up score on the observed score at baseline (i.e., IQ-20)) to produce a X_2_ residualized with respect to baseline. Specifically, the RDS approach was used to assess the effect of age-related changes in IQ across distinct time periods on brain-behavior relations, whilst the latter, RCA, was used to determine the subjects selected for this present study. Despite the initial warnings against the use of change scores [107], it has since been demonstrated that in some cases, analysis of change scores may provide an equal or even superior approach to exploring change over time. Namely, in investigations that utilize a randomized multivariate pretest-posttest design, or in studies that are vulnerable to confounding by response-shift effects (e.g. response contamination in self-report measures) [108,109]. Nonetheless, we briefly list the main objections against the use of difference scores in exploring change over time: 1. Difference scores are based on “imperfectly” measured pre- and posttest scores [109]. This imperfect reliability is commonly attributed to varying learning, personal or environmental influences that may affect the outcome measure at each sitting differentially. Thus, if difference scores are a combination of true change and change in any random error of measurement, the analysis of change scores may also be contaminated by these errors. 2. Raw differences tend to be (by construction) negatively correlated with baseline measures, potentially confounding the true relationship between the two measures of interest. 3. Raw difference scores are vulnerable to the well-known but poorly understood statistical artefact: regression toward the mean [110]. This phenomenon proposes that due to errors of measurement, an extreme score at baseline will be succeeded by a less extreme score at follow-up (i.e. one that is moving toward to the overall mean). Thus, difference scores originating from imperfectly observed measures will inherently provide an erroneous representation of “real” change, and 4. The discovery of spurious correlations that are due to active pre-existing differences at baseline [111].

Lastly, as the MDBC-1953 is a narrow-aged birth cohort, we are unable to compute moderation analyses to explore whether the significant relations identified in this study vary as a function of age, or if they demonstrate stability throughout the lifespan. However, a strength of the present study resides in its use of the EGD. That is, compared to traditional aging studies that are typically biased towards higher educated and better cognitively performing participants, the EGD increases the diversity in participant characteristics and with it the likelihood of discovering relations between variables that are more representative of the true population. Historically, the EGD – a common sampling procedure – has been used to accentuate statistical power of linear associations, facilitate the task of fitting a trend line to data, and reduce the costs associated with examining data from the full range of a variable [75]. In this specific study, the EGD was applied for two main reasons: First, to ensure that change in cognitive ability from early-adulthood to late midlife was sufficient to detect biological correlations. In this regard, the EGD is particularly useful in enhancing the variability in a measure of interest when only modest changes are expected – which is often the case in moderately sized, homogenous, healthy samples like the MDBC-1953. And second, by sampling subjects from the extremes of the change-in-IQ distribution we increased the cross-subject variability in cognitive change and other related variables (e.g. education level, occupation complexity, levels of motivation) ensuring that our sample was not biased towards higher educated, better cognitively performing, and motivated participants. Crucially however, the EGD does not undermine the validity of statistical significance: if the selection variable or related variables truly explain no variance, nominal false positive rates will be obtained. The approach can, however, bias parameter estimates of the selection variable or linked variables. In view of this, we ran several validation tests to explore the extent to which using this approach may have biased our findings. However, the result of the validation tests indicated that the significant relations observed at the whole-group level were not driven by group average differences (and hence not by the extreme-group-design either). Nevertheless, like all methods, the EGD is not without its own limitations, namely: artificial inflation of standardized effect sizes, power-enhancement for analyses of linear associations, increased vulnerability of extreme scores to the regression toward the mean phenomenon, low test-retest reliability, and unsuitability for testing nonlinear associations [75]. However, as the primary goal of this study was to detect brain-behavior relations and the extent to which they may be influenced by multiple, diverse aging-related covariates, we do not overstate the resultant effect-sizes, nor do we infer on how these relations may fluctuate over time. Rather, findings from this study can be used as a foundation for subsequent analyses exploring correlates of differential healthy aging trajectories. Furthermore, as the extant literature on healthy aging converge on findings that describe near linear declines in brain volume and cognitive ability we believe the limitation of the EGD to explore non-linear trends and associations therein is not a major shortcoming in this study.

## CONCLUSION

With the pressing personal and economic challenges presented by “top heavy” societies, this study offers a valuable approach for discovering potential age-related markers of early brain and behavior changes. Specifically, using data from a homogenous longitudinal prospective study, we report significant associations that link broad-brush and specific brain and cognitive measures to each other and to a range of heterogeneous age-related covariates. Specifically, CCA identified individual variations in brain structural patterns that were not only significantly associated to each other but also related to individual differences in behavior. Future longitudinal studies using a larger sample size, > 2 measurement occasions, and a broader selection of potentially relevant age-related covariates (e.g. fMRI data [112], markers of oxidative stress [23], inflammatory processes [113], immuno-senescence [114], telomere attrition [115], hormonal dysregulation [10], and brain metabolites [10]) are likely to explain larger portions of the unexplained variance in healthy aging trajectories, ultimately improving early intervention targets and with it, the quality of life for older adults.

## MATERIALS AND METHODS

### Participants: extreme group design (n=1,985)

Details regarding the subject selection criteria used for the imaging sub-study have been previously reported [112]. This study has also been registered at clinicaltrials.gov (NCT03290040). In summary, using youth and late midlife intelligence quotient (IQ) scores, subjects were selected based on their estimated change in mental ability as part of an “extreme group design” (EGD) [116]. Specifically, the two well-validated tests, the Børge Priens Test (BP) [117] and Intelligenz-Struktur-Test 2000 R (IST) [118] were taken at ages ~20 (IQ-20) and ~57 (IQ-57) respectively. The BP test was used as part of a military draft board assessment on a total of n=11,532 MDBC-1953 subjects, whilst the latter reassessment test, IST, was administered in 2009-2011 by the Copenhagen Ageing and late midlife Biobank Project (CAMB) and included n=1,985 members [14]. Both examinations comprise subtests that assess aspects of verbal and arithmetic intelligence (e.g. numerical series and verbal analogies), and thus are similarly structured and comparable. Since the cognitive change between these time-points was based on two different instruments, a change score was derived with a linear regression analysis of IQ-57 (IST-2000 R) on IQ-20 (BP) using the whole population of 1,985 CAMB subjects. IQ-20 explained R^2^=50.4% of variance in IQ-57 (beta=0.71, p<0.0001), and we used each subject’s standardized residual about the regression line as a measure of their change in IQ across time, Supplementary Figure 1. To avoid the effects of extreme test scores, subjects with absolute standardized residuals ±3 were omitted. Finally, the remaining members were classified into two subgroups pertaining to the degree of cognitive change observed from early-adulthood: subgroup A = improvers and subgroup B = decliners.

### Participants: present study (n=193)

**Figure 5:**
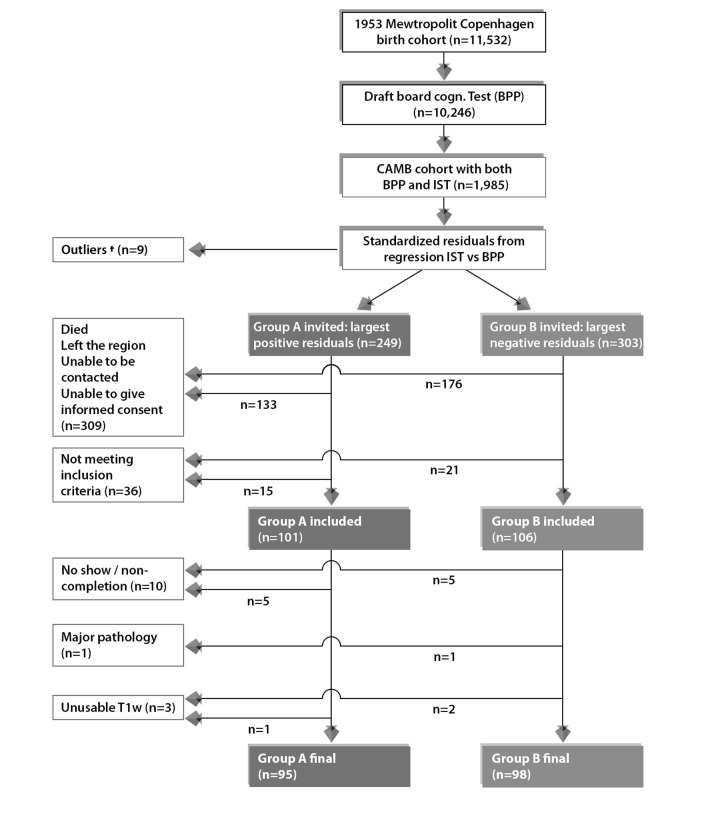
**Subject selection process for current study from the 1953 Metropolit Danish Male Birth Cohort based on the “Extreme Group Design”.** To avoid effects of extreme test scores, subjects with standardized residuals ±3 were omitted, defined as here as †. The final sample size for the current study includes n=193 subjects consisting of n=95 improvers and n=98 decliners in cognitive function from youth to late midlife.

Acquisition of imaging and non-imaging data for this study was carried out in 2010-2013 (subject age 57±0.8 years). During this period, a total number of n=552 subjects were invited to participate, and of these, n=243 accepted their invitation and proceeded to the data acquisition stage. Here, subjects suffering from alcohol or drug abuse comorbid with cognitive impairment, psychiatric or neurological disease, and contraindications to MRI were identified and eliminated from further investigation (n=36). Out of the remaining eligible respondents, a further 14 subjects were removed due to imaging-related contraindications or having T1-weighted (T1w) structural brain images that were unusable, leaving a total of n=193 subjects data that were used in this present study (subgroup A: n=95; subgroup B: n=98), Figure 5. Although there was a small range of ages during data acquisition, in general we refer to the ages of participants as 11 (W-11), 20 (W-20), 57 (W-57), and 63 (W-63) years. Data pertaining to W-63 (i.e. the second late-midlife data sweep) includes both brain imaging and behavioral data and is subject to a subsequent report. This study was approved by the local ethical committee (De Videnskabsetiske Komiteer for Region Hovedstaden) and registered by the Danish Data Protection Agency. All participants provided written informed consent.

### Data

### *Neuropsychological assessment*

An in-depth series of neuropsychological tests were administered on the same day as the brain-MRI acquisition. Global cognitive function was assessed with the mini-mental state examination (MMSE) and Addenbrooke’s cognitive examination (ACE). The Cambridge Neuropsychological Test Automated Battery (CANTAB) was administered to evaluate cognitive ability across the following cognitive domains: learning and memory (spatial and pattern recognition, paired associates learning), executive function (planning), and attention and reaction time [119]. Furthermore, we include measures of intelligence acquired at W-11 (IQ-11) and W-63 (IQ-63). IQ-11 was assessed using the Härnquist test battery and consists of three subtests evaluating spatial, numerical and verbal intelligence [120]. Similar, to IQ-57, IQ-63 was also assessed using the IST 2000R test at the latest 5-year late-midlife follow up. Although we are unaware of any studies that evaluate the validity of the Härnquist test, previous studies using subjects from the MDBC-1953 have reported strong correlations between measures of IQ-11, IQ-20 and IQ-57 [14]. Furthermore, the BP test has previously shown strong correlations with the Wechsler Adult Intelligence Scale [121]. In total, we include 31 measures of cognitive performance. See Table 1 for a list of these variables and the study sample characteristics.

### *Demographic, health and lifestyle assessment*

We evaluate the effect of environmental, lifestyle behaviors and biological factors, both positive and negative, on individual differences observed in brain structure and cognitive performance. These include: Major Depression Inventory (MDI) [122], Pittsburgh Sleep Quality Index (PSQI) [123], Multidimensional Fatigue Inventory (MFI-20) [124], and a range of demographic (social and biological), vascular risk factors (VRFs), general health and biological samples for biomarker analyses. In total, we report 39 measures of other (non-imaging) variables, which together with the neuropsychological data described above are referred to as non-imaging derived phenotypes (non-IDPs) or behavioral data. Finally, to assist the interpretation of results, non-IDPs were divided into four subdomains: cognitive, demographic (social and biological), health and lifestyle. Tables 1-4 provide a list of these variables, how they were assessed and study sample characteristics.

### MRI data acquisition

All subjects underwent whole-brain MRI scanning using a 3.0 T Philips Intera Achieva (Philips Medical Systems, Best, the Netherlands), with a 32-channel phased-array head coil. During resting conditions the following sequences were acquired in all participants: 1. anatomical high-resolution 3D T1-weighted (T1w) images using a gradient echo sequence (TR/TE = 6.9/700 ms; flip angle = 9˚; voxel size = 1.1 x 1.1 x 1.1 mm^3^), 2. T2-weighted (T2w) (TR/TE = 1300/12 ms; flip angle = 90˚, 32 slices, voxel size 1.8 x 1.8 x 9.5 mm), 3. T2w FLAIR (T2w-FLAIR) images (Fluid Attenuated Inversion Recovery) using turbo spin echo sequence (TR/TE = 11000/125 ms; ; flip angle = 90˚, 6 slices, voxel size = 0.45 x 0.45 x 4.5 mm), and 4. diffusion weighted images (dMRI) (TR/TE = 9729/55; matrix =112 x 110 x 60; voxel size = 2 x 2.04 x 2 mm^3^) utilizing a single spin-echo echo-planar imaging sequence. For each dMRI scan, 33 images were acquired: 1 image with no diffusion sensitization (b=0 image), and 32 diffusion-weighted images (b = 1000s/mm^2^). Using varying orthogonal views, all T1w, T2w, T2w-FLAIR and dMRI images were visually inspected in their raw state for bias field corruption, excessive motion, and other potential artifacts. Lastly, although not used in this study, the MRI protocol also includes task and resting functional MRI (fMRI) imaging.

### Image analysis pipeline

In order to facilitate future meta-analyses and replication studies, we used the UKB image processing pipeline on our raw (non-processed) imaging data [125]. A strength of this pipeline is that it ameliorates the loss of statistical power by reducing the number of multiple comparisons made (compared with voxel-wise testing) and increasing signal-to-noise ratio (SNR) by replacing voxel-wise measures with ROI (region of interest) averages obtained through alignment to a standard coordinate system. For a detailed overview of the imaging pipeline see [126].

In brief, we extracted 453 brain-imaging biomarkers (i.e., a parsimonious set of biologically meaningful measures derived from multiple imaging modalities) that best capture the differential aging processes and neuropathology observed in a healthy aging population. Subsequently, we categorized the extracted summary measures into 5 groups (T1w-SIENAX, T1w-FIRST, T1w-FAST, T2w-FLAIR-BIANCA, and dMRI-TBSS) to reflect the MRI modality and image processing tool applied to estimate each measure, Table 7. Specifically, we used T1w structural data as the reference image to calculate cross-subject and cross-modality alignments required to process all other brain modalities. Additionally, T1w data is used to obtain estimates of both specific brain structures (primarily sub-cortically), and volumes of major tissue types of the whole brain (i.e., grey matter (GM), white matter (WM) and ventricular cerebrospinal fluid (CSF)), which is subsequently used to provide robust biomarkers of global and local brain atrophy [95]. See Supplementary Material (“T1w Pipeline”) and Supplementary Figure 2 for further details. Next, we use T2w-FLAIR imaging to derive estimates of focal hyperintensities in WM which are typically indicative of inflammatory disease, cerebrovascular pathology, demyelination, trauma, or other neural insults [36,127]. We also use dMRI to provide an index of the density and structural integrity of WM microstructure in vivo (e.g., neurites and axonal structures) based on the principle of random diffusion of water molecules within cellular compartments [40,128]. Specifically, it has been shown that dMRI may be able to measure changes in normal appearing WM (NAWM) before these can be identified using more traditional MRI techniques e.g. T1w or T2w [39,76,93,129]. Lastly, in a previous sub-study, phase contrast mapping (PCM) was utilized to measure volume flow in basilar and the internal carotid arteries. Here, total blood flow to brain size was normalized and used to derive mean global cerebral blood flow [112,130]. For a detailed overview of the image processing pipeline refer to Supplementary Material and Figure 2, Figure 3A, Figure 3BFigure 4AFigure 4B.

**Table 7:** List of imaging-derived phenotypes (IDPs).

MRI MODALITY	PROCESSING TOOL		FUNCTION	DESCRIPTION		N
				
**T1-w ** ** ** ** ** ** ** ** **	FAST (FRMIB’s Automated Segmentation Tool)		Discrete and probabilistic segmentation of CSF, GM, & WM	Total volume of GM of cerebellum and non-cerebellum ROIs using GM partial volume estimates from FAST*		**139**
			
	FIRST (FRMIB’s Integrated Registration and Segmentation Tool)		Subcortical GM structure segmentation	Lateralised brain structures + brain stem		**15**
			
	SIENAX		Estimation of brain tissue volume (cross-sectional)	Global brain tissue volume (unnormalized and normalized for head size)		**10**
				
**T2-w (FLAIR) **	BIANCA (Brain Intensity Abnormality Classification Algorithm)		Quantification of total WMH load	WMH load		**1**
** **						
**Diffusion **	TBSS (Tract Based Spatial Statistics)		Diffusivity estimates within 48 major WM tracts	Local diffusion properties reflecting integrity of microstructural WM tissue		**288**
**TOTAL IDPS = 453**						
				

List of imaging modalities and processing tools employed (column 1 and 2), followed by the corresponding function of each processing tool (column 3), description of the brain measure(s) extracted (column 4), and total number of IDPs estimated (column 5) using the UK Biobank (UKB) image-processing pipeline. (Abbreviations: FLAIR = fluid attenuated inversion recovery, CSF = cerebrospinal fluid, GM = grey matter, WM = white matter, WMH = white matter hyperintensity load, ROIs = regions-of-interest). Total number of IDP measures included, n=453. *The 139 ROIs are defined by a combination of parcellations from the following atlases: Harvard Oxford cortical and subcortical atlases, and Diedrichsen cerebellar atlas, for further details see Supplementary Material "T1w Pipeline"  and Figure 2.

### Statistical analysis

**Figure 6:**
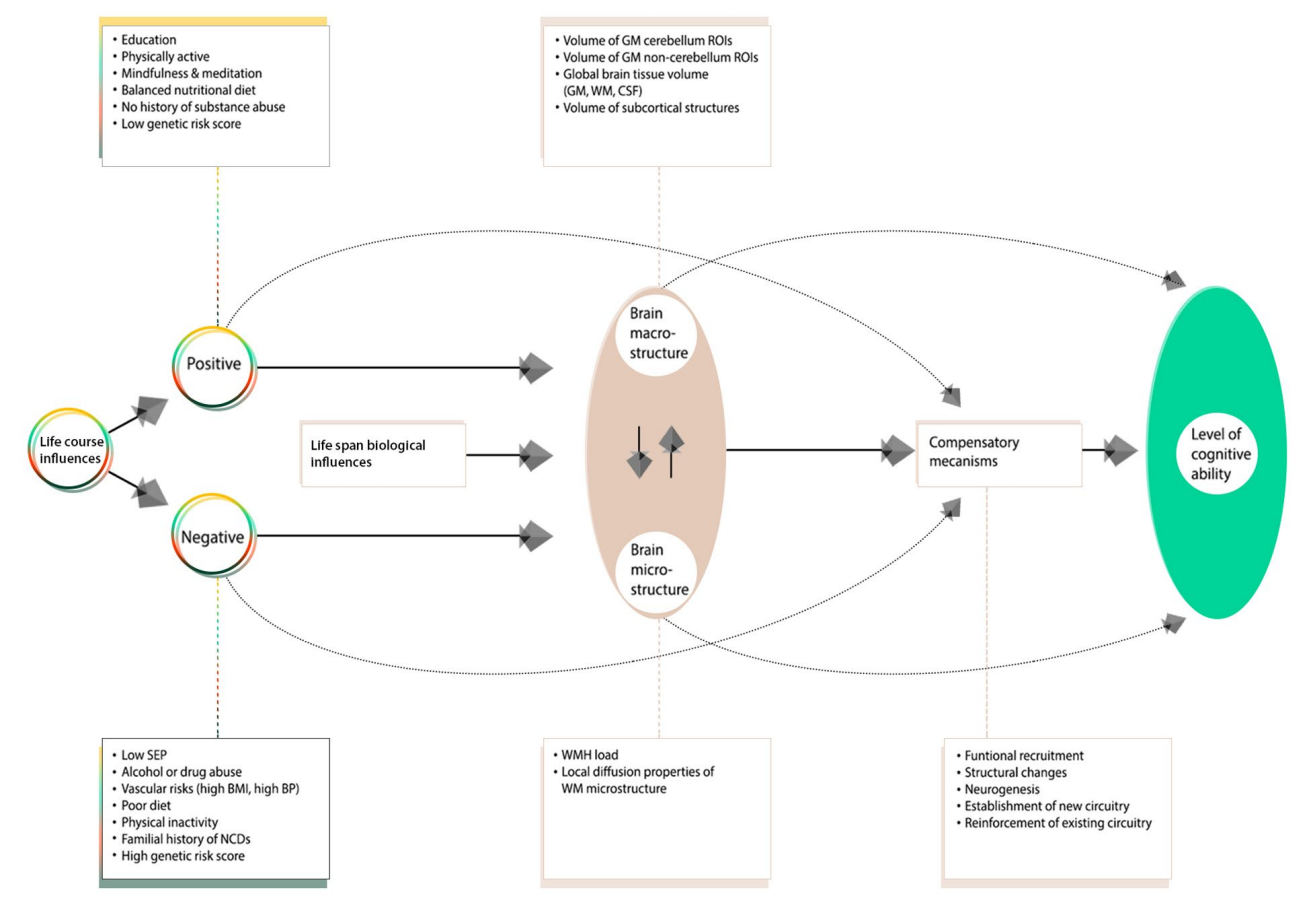
**A revised conceptual model of the Scaffolding Theory of Aging and Cognition (STAC-r**). The original STAC model offered a theory that only accounted for individual variations in cognitive performance observed at one time point – later adulthood, with “aging” as the primary input to the model. In view of the latest developments within the field of aging research, the contribution of lifespan experiences (positive and negative) on later-life brain and cognitive health has led to a variant of the model that incorporates the cumulative effects of lifespan environmental and biological experiences on neurocognitive aging. In this adapted figure, we only present biomarkers and traits investigated in this study. Specifically, Figure 6 offers a pathway of how the additive bidirectional lifespan experiences may contribute to the differential age-related trajectories of health. We distinguish between influences that act to preserve neural integrity (“positive influences”) versus those that are implicated in its demise (“negative influences”).

To investigate whether brain IDPs contain relevant between-subject information pertaining to sets of other (non-imaging) MDBC-1953 variables, we used both univariate (Pearson correlations) and multivariate statistics (canonical correlation analysis (CCA) [131], both analyses adjusted for a number of confound variables. Figure 6 visualizes the selection of candidate determinants included in this study and a hypothesized pathway of how their additive bidirectional effects may lead to an alteration in brain and behavior. In this study, we distinguish between influences that may act to preserve neural integrity (positive influences) versus those that are implicated in its demise (negative influences).

### *Whole-group adjusted univariate associations*

We applied Pearson’s correlations to study the relation between each of the 453 brain IDPs to each of the 70 non-IDP variables and each of the 31 cognitive measures to each of the (other) 39 non-IDP variables extracted from the MDBC database (full set of IDP x non-IDP estimates: 453 x 70; full set of cognitive x all (other) non-IDP estimates: 31 x 39). The motivation behind this approach was to first identify significant simple pairwise associations between each brain and non-brain measure and each cognitive and other non-IDP measure, before comparing these results to those from more complicated multivariate techniques accountable for the synergistic effect of multiple variables. In total, two variants of Manhattan plots are used to display the significance (-log10 P-values) of Pearson’s correlations for IDPs x non-IDPs (31,710 values) and cognition x all (other) non-IDPs (1209 values).

To reduce the influence of potential outliers and increase the reliability of associations made, we applied rank-based inverse Gaussian transformation (quantile normalization) to enforce Gassianity for each of the IDPs, non-IDPs, and confound variables (see below). For feeding into CCA (but not needed for univariate associations), we then applied an iterative PCA algorithm (based on the soft shrinkage of eigenvalues) to impute missing data values until convergence [132]. Finally, four confound variables were created relating to effects that may trouble the interpretation of computed correlations: absolute motion during MRI, relative motion during MRI, head size, and age. The confounds were regressed out of all IDP and non-IDP variables. To account for multiplicity, we assessed the strength of significance against two different types of multiple testing correction, controlling the familywise error rate (FWE) via Bonferroni and the false discovery rate (FDR) [133]. However, as FDR correction did not identify any additional tests already marked significant by Bonferroni, Figure 1 and Supplementary Figure 7A-7B only show Bonferroni correction.

### *Validation test: subgroup univariate associations*

In order to assess the impact of the EGD, we separately compute univariate associations between each IDP and non-IDP measure for subgroup A (“improvers”) and subgroup B (“decliners”) subjects. Each subanalysis should be free of any spurious associations driven by average group differences in cognitive level (i.e., an example of Simpson’s paradox [134], whereby suboptimal pooling across variables such as cognitive level can potentially generate misleading associations).

### *Whole-group univariate associations: adjusted for change in IQ (C∆)*

We compute univariate associations between each IDP and non-IDP measure and between each cognitive and all (other) non-IDP measures after adjusting for change in normalized IQ score pertaining to cognitive tests administered during early-life, youth and late-midlife. Specifically, change in IQ was estimated using the raw difference score (RDS) approach (i.e. subtracting normalized post-test scores from normalized pre-test scores). Here we explore the effect of cognitive change on brain-behavior univariate relations with a specific focus on how individual differences in cognitive change may contribute to the brain-cognition relations observed in late-midlife. The three cognitive change variables estimated include: C∆1 = IQ-57 – IQ-20, C∆2 = IQ-63 – IQ-57, and C∆3 = IQ-20 – IQ-11.

### *Whole-group adjusted multivariate associations*

**Figure 7:**
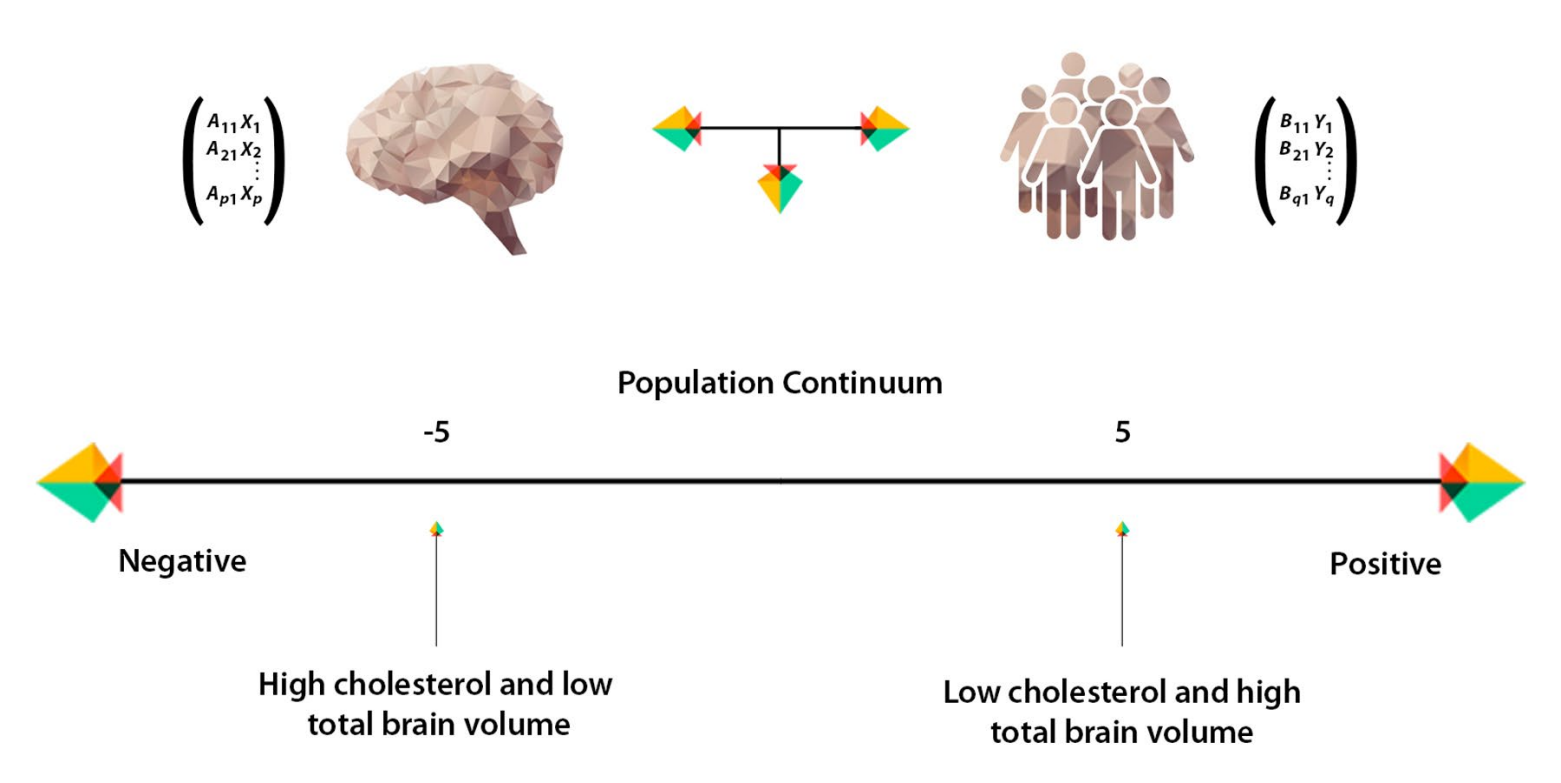
**Population continuum.** CCA estimates a “population continuum” of covariation across subjects that jointly characterizes brain imaging and other (non-imaging) data through a single axis. We can describe each subject’s relation to this axis by assessing the value and polarity assigned to their individual CCA-derived subject weight and further evaluating how this value is related to the observed IDP and non-IDP measures. In this example, subjects with negative subject weights are characterized by high total cholesterol levels and low total brain volume, whereas subjects assigned with positive subject weights are characterized by low total cholesterol levels and higher total brain volume.

To capture patterns of age-associated relations in multiple IDP and non-IDP measures simultaneously we applied CCA, Figure 7.

For the CCA, we adopted a similar approach as described previously [78,125]. In short, CCA was computed (canoncorr; MATLAB 2014a) following the model: U = AX and V = BY; where X represents the set of IDPs, Y is the set of non-IDPs, and A and B are optimized to maximize the correlation between each canonical variate pair, U and V [131]. The magnitude of the relationship between each variate pair is reflected by the canonical correlation coefficient (R_c_), an indicator of how strongly the estimate of population covariation is reflected in both IDP and non-IDPs datasets. Intuitively, we can think of CCA as identifying two latent variables, U_i_ and V_i,_ from a specific linear combination of weighted MRI-derived brain measures that are most strongly associated to a specific linear combination of weighted non-imaging measures, Supplementary Figure 5A-5B.

IDP and non-IDP datasets for CCA analysis were prepared using the procedure described in "Whole-Group Adjusted Univariate Associations". This resulted in a brain-IDP matrix of size 193 x 453 (subjects × IDPs) and a non-IDP matrix of size 193 × 70 (subjects x non-IDPs). Typically, these datasets are the inputs fed into the CCA algorithm. However, to reduce overfitting (i.e., tending towards a rank-deficient CCA solution), prior to CCA we separately reduced the dimensionality of each dataset using PCA. Specifically, after accounting for missing data as before, we compressed the size of each matrix along the respective phenotype dimension to the top 30 subject-eigenvectors which accounted for ~75% of the total variance in our datasets (70.1% for IDPs, 77.6% for non-IDPs). The final dimension of each matrix fed into CCA was therefore 193 x 30 (subjects x PCA-derived components), with an output of 30 CCA modes estimated.

Statistical significance of the modes estimated was determined using 10,000 permutations of rows of one matrix relative to another. CCA was then re-run after each permutation and the respective r-values for each permuted CCA mode was estimated. Each observed canonical correlation r is compared to the null permutation distribution of the largest canonical correlation, creating familywise error p-values corrected for searching over all 30 canonical correlation dimensions.

### Post-hoc correlations

To relate the CCA mode estimated back to the observed IDP and non-IDP variables, we perform post-hoc correlations. This is achieved by computing the correlation between each original (observed) variable and the CCA-derived canonical variate weights (U or V), Supplementary Figure 6. This approach is analogous to the computation of factor loadings in factor analysis, and are also known as canonical structure coefficients. Generally, variables with larger loadings indicate greater association with a CCA mode. Finally, to formally assess the degree of similarity in the CCA subgroup analysis, we provide a coefficient of factor congruence between groups (i.e. improvers vs decliners) for the IDPs and non-IDPs.

## Supplementary Material

Supplementary Material
