# Supplementary material for: Discovering markers of healthy aging: a prospective study in a Danish male birth cohort
Source: Aging (Albany NY). 2019 Aug 26;11(16):5943–74. doi: 10.18632/aging.102151 (PMC6738442; doi:10.18632/aging.102151)
Supplement: Supplementary Material [file aging-11-102151-s001.pdf]

SUPPLEMENTARY MATERIALS

Supplementary Methods

Image Preprocessing Pipeline

The DMBC-1953 imaging data were minimally preprocessed using tools from FSL [1] following UKB image preprocessing guidelines. In brief, preprocessing of T1w images included: reduction in the field-of-view (FoV), brain extraction (using FSL tool BET [2]), linear alignment to the MNI standard-space brain template [3, 4], followed by a nonlinear alignment (FNIRT) to the MNI standard space to achieve maximum agreement across participants irrespective of the large individual differences in brain structure [5].The latter non-linear warp is of great importance as it is this inverted transformation that is applied in multiple subsequent steps of the processing pipeline. Preprocessing of T2w-FLAIR images follow a similar procedure to T1w images but with a few minor differences. Namely, brain extraction is not directly applied to the original T2w images; instead, T2w images are linearly aligned to T1w space using FLIRT, and the subsequent transform is then concatenated with the T1w-derived transforms generating T2w-FLAIR image versions that are in MNI standard space and T1w native space. In the final steps, we apply the bias field estimated from the T1w pipeline (i.e. derived from FAST) to the transformed T2w-FLAR images to remove B1 field inhomogeneity's.

T1w Pipeline

Supplementary Figure 2 provides a visualization of the T1w processing pipeline. In brief, the T1w pipeline incorporates both artefact removal (i.e. removal of non-brain tissue above and below the head using FSL tools robust FoV and BET) and linear registration (i.e. alignment to the MNI152 "non-linear 6th generation" standard-space T1 template). This is followed by a non-linear registration to the standard-space MNI152 brain mask using FNIRT [5]. Ultimately, for each subject, the T1w pipeline generates one standard T1w brain mask in native space which is necessary for estimating whole brain volumes of major tissue types and sub-cortical structures. Specifically, this is achieved using the inverse of the nonlinear T1w-to-MNI alignment warp to transform a standard-space brain mask into native T1w space. As T1 structural images are used as a reference for all other imaging modalities, all estimated transformations are applied in a single step as late in the pipeline as possible avoiding additional interpolation-related smoothing. Additionally, the resulting T1w brain mask from phase 4 is used to create a more accurate brain-extraction, replacing the original brain extraction from phase 1. Finally, tissue type segmentation is performed using FAST [6], and the resulting data are then used to create the T1w-derived IDP summary measures. In total, we estimate: total volume of GM (derived from GM partial volume estimates modelled by FAST) within 139 GM ROIs defined by a combination of parcellations from the Harvard Oxford cortical and subcortical atlases and Diedrichsen cerebellar atlas [7, 8], 15 sub-cortical structures (shapes and volumes) modelled by FIRST [9], 10 (normalized and unnormalized for head size) global brain volume measures of total WM volume, total GM volume, total brain volume (GM + WM), ventricular (non-peripheral) CSF volume, and peripheral cortical GM volume modelled by SIENAX [10], part of the FSL toolbox [11], Table 6. Note, in order to obtain the GM volume of the 139 ROIs, we inverted the nonlinear registration to standard space, and used this to warp a cortical atlas of 139 ROIs into native T1 space. Within each ROI we then summed the total GM, as estimated via the FAST GM partial volume estimates.

T2w-FLAIR Pipeline and the Brain Intensity Abnormality Classification Algorithm tool (BIANCA)

Supplementary Figure 3 provides a visualization of the T2w-FLAIR pipeline. As previously mentioned, we apply the nonlinear T1w-to-MNI152 warp to transform T2w-FLAIR images to MNI152 space. This step supports the format of input images required for other combined analyses, namely, WMH (or white matter lesion) segmentation using the Brain Intensity Abnormality Classification Algorithm tool (BIANCA) [12]. As shown in Figure 3, a linear registration is applied to the original T2w-FLAIR image to transform the T2w-FLAIR into T1 space. Next, using the previously calculated transformation warp from T1 to MNI152, we create an MNI152 version of T2w-FLAIR images. Finally, we apply the estimated bias field previously calculated by FAST in the T1w pipeline to correct for residual bias field inhomogeneities in the T2w-FLAIR image.

BIANCA is a fully automated, supervised method for WMH detection, based on the k-nearest neighbor algorithm which estimates the voxel-wise probability of WMH prevalence. Specifically, using T2w-FLAIR data (in T1w space), unbiased T1w data, T2-FLAIR-to-MNI warp and a WM mask that

excludes cortical GM, subcortical structures and spurious hyperintensities (e.g., derived from CSF pulsation artefacts), BIANCA generates one estimate of total volume of WMHs from all voxels that exceed a threshold of 0.9.

dMRI Preprocessing

Supplementary Figure 4 provides a visualization of the dMRI pipeline. In brief, each raw dMRI volume is affine-aligned to the reference (b=0) image to correct for head motion and eddy current distortions using FSL's eddy tool [13]. The corrected dMRI data are then brain extracted. The resulting data are then fed as input to tract-skeleton (TBSS) [14] processing to extract estimates of diffusivity measures contained within 48 standard-space WM tract ROIs [15].

TBSS in more detail: We begin by fitting a single diffusion tensor model (ellipsoid) to each voxel in the corrected diffusion volumes using dtifit [14], part of FSL Diffusion Toolbox [11]. This process describes voxel-wise diffusion orientations pertaining to the magnitude and directionality of water displacement across brain tissue. Traditionally, diffusion of water molecules in brain tissue is described as isotropic in GM (i.e., the magnitude of diffusion is invariant in each direction) and more anisotropic (i.e., restricted to the major axis) in WM. The barriers of water displacement across the brain tissue have been attributed to the presence of myelin sheaths, axonal filaments and microfilaments [16]. The following sources provide detailed discussions regarding the principles and biophysics of DTI [17-19]. Specifically, for each voxel, the diffusion tensor predicts the pattern of diffusion using the axes (eigenvectors) of an ellipsoid to provide estimates of the major and two further (orthogonal) minor axes. Using the tensor-model derived eigenvalues $\lambda 1$, $\lambda 2$ and $\lambda 3$ (i.e. estimates of the rate of diffusion along each eigenvector), we calculate the most frequently used DTI summary measures, fractional anisotropy (FA) [20], mode of anisotropy (MO) [21], and mean diffusivity (MD) [20, 22]. Furthermore, to deepen our understanding of the biophysical mechanisms of water displacement across the brain tissue we also include individual eigenvalues, $\lambda 1, \lambda 2, \lambda 3$, to elucidate the role of axial ($\lambda_{ax} = \lambda 1$) and radial (λ_{rad} =mean of $\lambda 2$, $\lambda 3$) diffusivity changes as potential markers of WM integrity in ageing [23, 24]. Specifically, FA is a normalized scaler index, ranging for 0 (isotropic) to 1 (anisotropic), that reflects the total magnitude of anisotropic (directional/restricted) diffusion. In age- or disease-related neurodegeneration a decline in FA is conventionally reported to indicate various microstructural processes including demyelination, axonal degradation or gliosis [25]. The tensor index, MO, provides further details complementary to FA that characterizes the type of anisotropic diffusion using variations in the shape of the tensor model. This value can range from -1 to +1 describing a more planar/disc-like shape (i.e., in crossing fibers regions where axes one and two are of similar densities $\lambda 1 \sim \lambda 2 > \lambda 3$) to one that is more linear/cigar-like (i.e., regions where one axis predominates $\lambda 1 > \lambda 2 \sim \lambda 3$). An increase in MO (i.e., a planar-to-linear transformation), with concomitant increases in FA, has been attributed to the selective degeneration of secondary WM tracts following a primary lesion [26]. Thus, caution must be applied when interpreting FA and MO measures in brain regions that contain intersecting WM tracts. Specifically, it has been demonstrated that selective degenerative of WM tracts in crossing fiber regions can affect fibers arranged in one direction (e.g. cognitive-associated association fibers) but not those transverse to it (e.g. motor-related projection fibers) [27]. This results in the "spared" fibers of this sub-region defining the newly acclaimed primary eigenvector, and with it creating a misleading increase in both FA and MO. Contrary to anisotropy diffusion, MD provides a measure of the average rate of diffusion across all three orthogonal eigenvectors and generally serves as a sensitive but general indicator of neurodegeneration. Specially, an increase in MD is commonly associated to decreases in membrane density or increases in interstitial or extracellular fluid [20, 22]. Lastly, component measures λ_{ax} and λ_{rad} reflect the mean diffusion coefficient of water parallel or perpendicular to the primary fiber orientation respectively. To date, decreases in λ_{ax} have been reported to reflect axonal damage, while independent increases in λ_{rad} have been implicated in myelin-specific degeneration [24, 28-30]. However, deviations from this pattern have also been observed [25-27, 31] and interpretation of raw eigenvalues should be used in collaboration with other commonly used DTI metrics.

TBSS permits between-subject comparisons of WM pathways by aligning each subject's FA image to a standard target image, thus rendering all FA maps into the same 3D coordinate space. This is achieved through non-linear alignment of the FA image (derived from DTIFIT) onto a standard-space FA image using a high dimensional FNIRT-based warp [32]. The resulting high-dimensional FNIRT-based

warp is also applied to all other DTI-derived output maps (i.e. MO, MD, λ_{ax}, λ_{rad}). These data are then averaged to create a study-specific standard mean FA, MO, MD, λ_{ax}, and λ_{rad} map which are subsequently skeletonized - and for FA and MO thresholded at FA > 0.2 to include only highly anisotropic anatomy. Lastly, the skeletonized images are averaged within a set of 48 standard-space WM tract masks defined by the John Hopkins University (JHU) dMRI-based WM tractography atlas [33]. In total, we estimate n=288 diffusivity measures per subject.

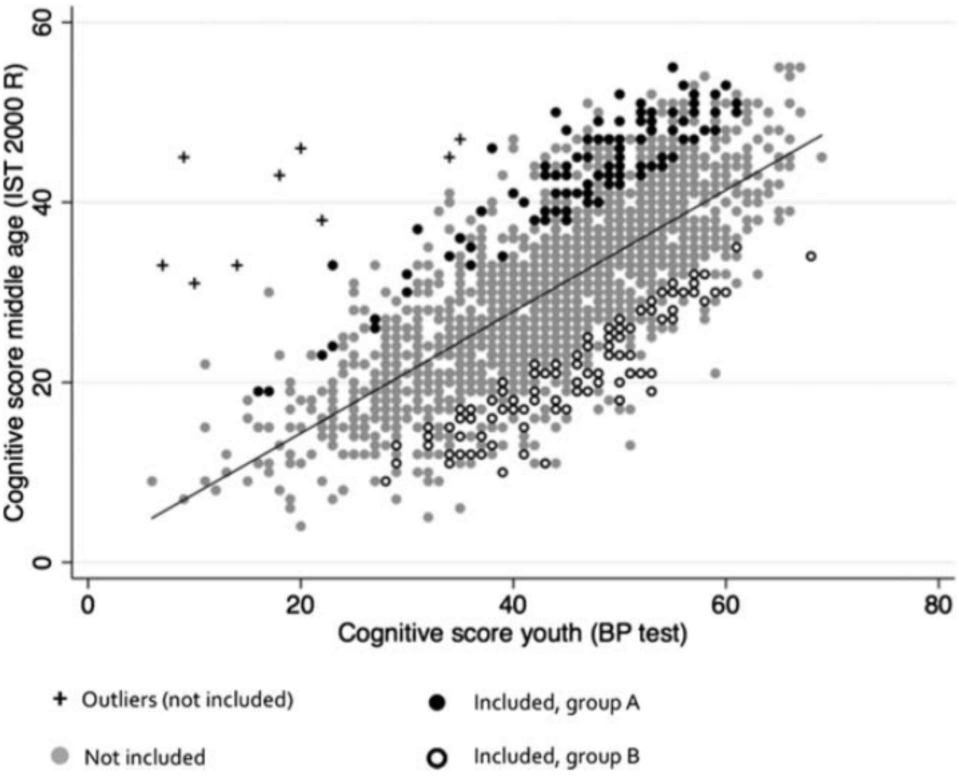

Supplementary Figure 1. Subject selection using an "Extreme Group Design" (EGD). Subjects were recruited based on a change score derived using a linear regression analyses of cognitive ability at age ~57, IQ-57 (IST2000-R test), on cognitive ability at age ~20, IQ-20 (BP test). The regression model estimates the expected performance at late midlife given each subjects BP score using the whole population of n=1985 CAMB participants. IQ-20 explained R^2=50.4% of variance in IQ-57, and subjects with the highest (±) absolute residual score were selected. For this study, we included group A (positive residuals) and group B (negative residuals) subjects.

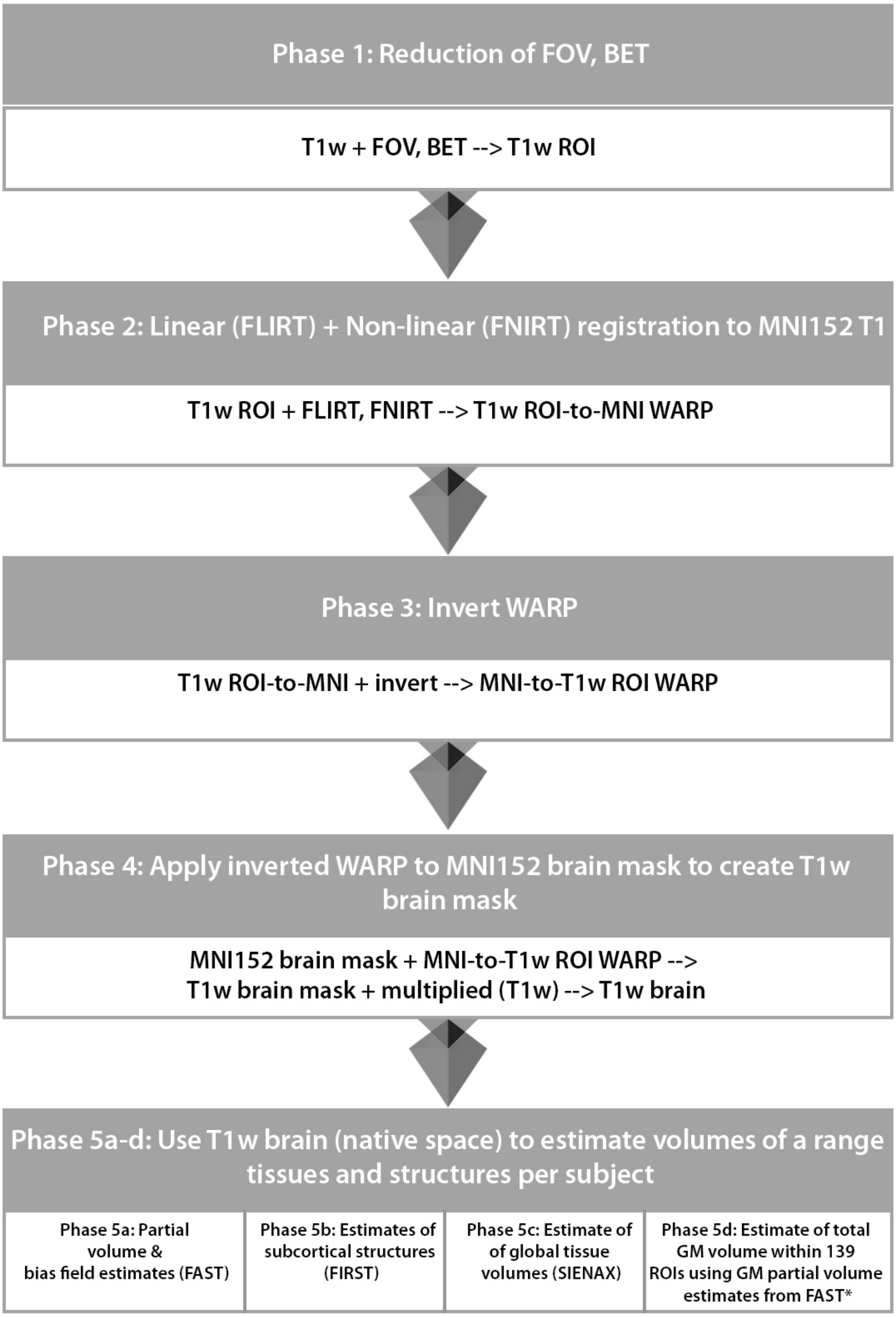

Supplementary Figure 2. Flowchart of T1w processing pipeline. *In order to obtain the GM volume measures of the 139 ROIs, we inverted the nonlinear registration to standard space, and used this to warp a cortical atlas of 139 ROIs into native T1 space. Within each ROI we then summed the total GM, as estimated via the FAST GM partial volume estimates. The 139 ROIs were defined by a combination of parcellations from the following atlases: Harvard Oxford cortical and subcortical atlases, and Diedrichsen cerebellar atlas.

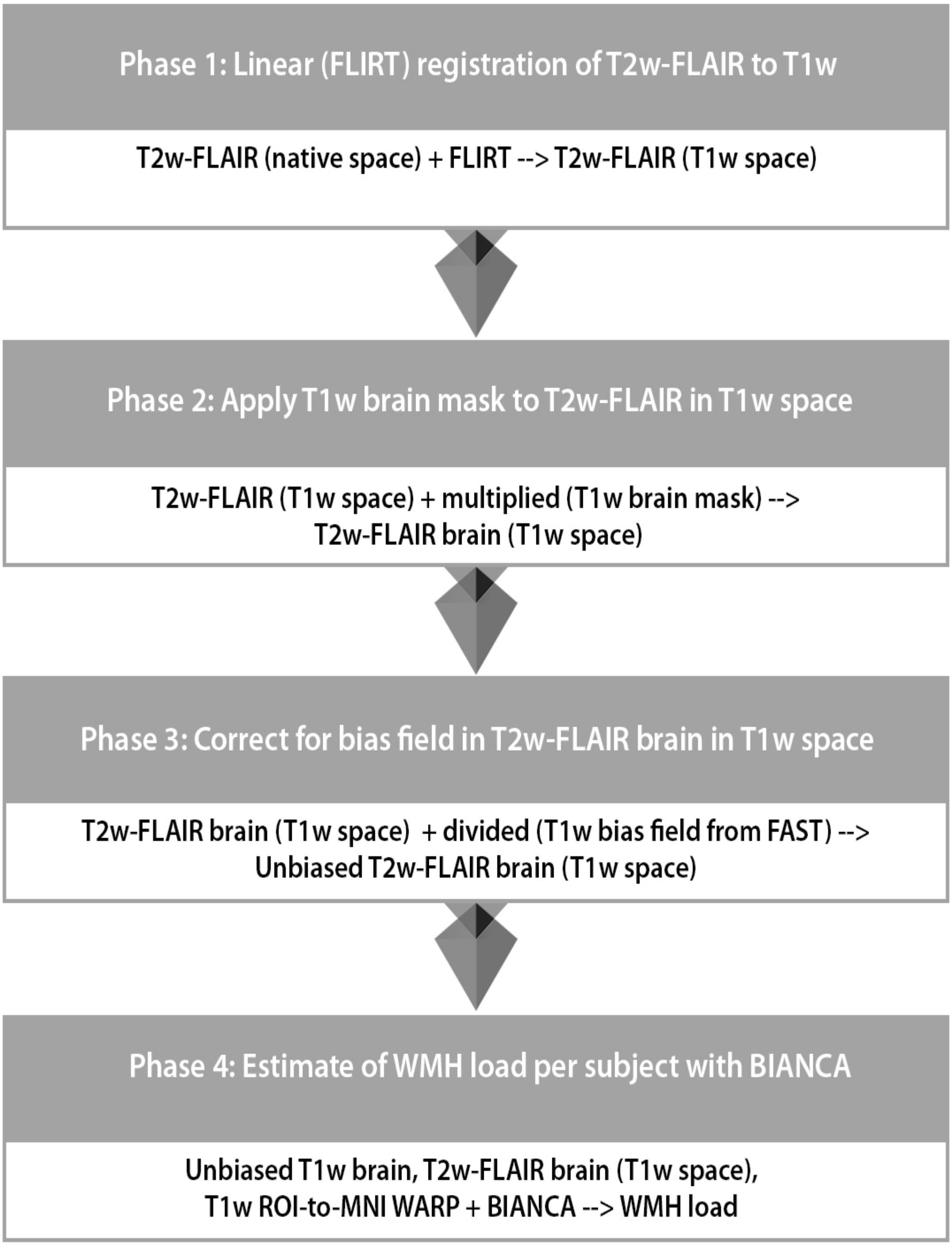

Supplementary Figure 3. Flowchart of T2w-FLAIR processing pipeline.

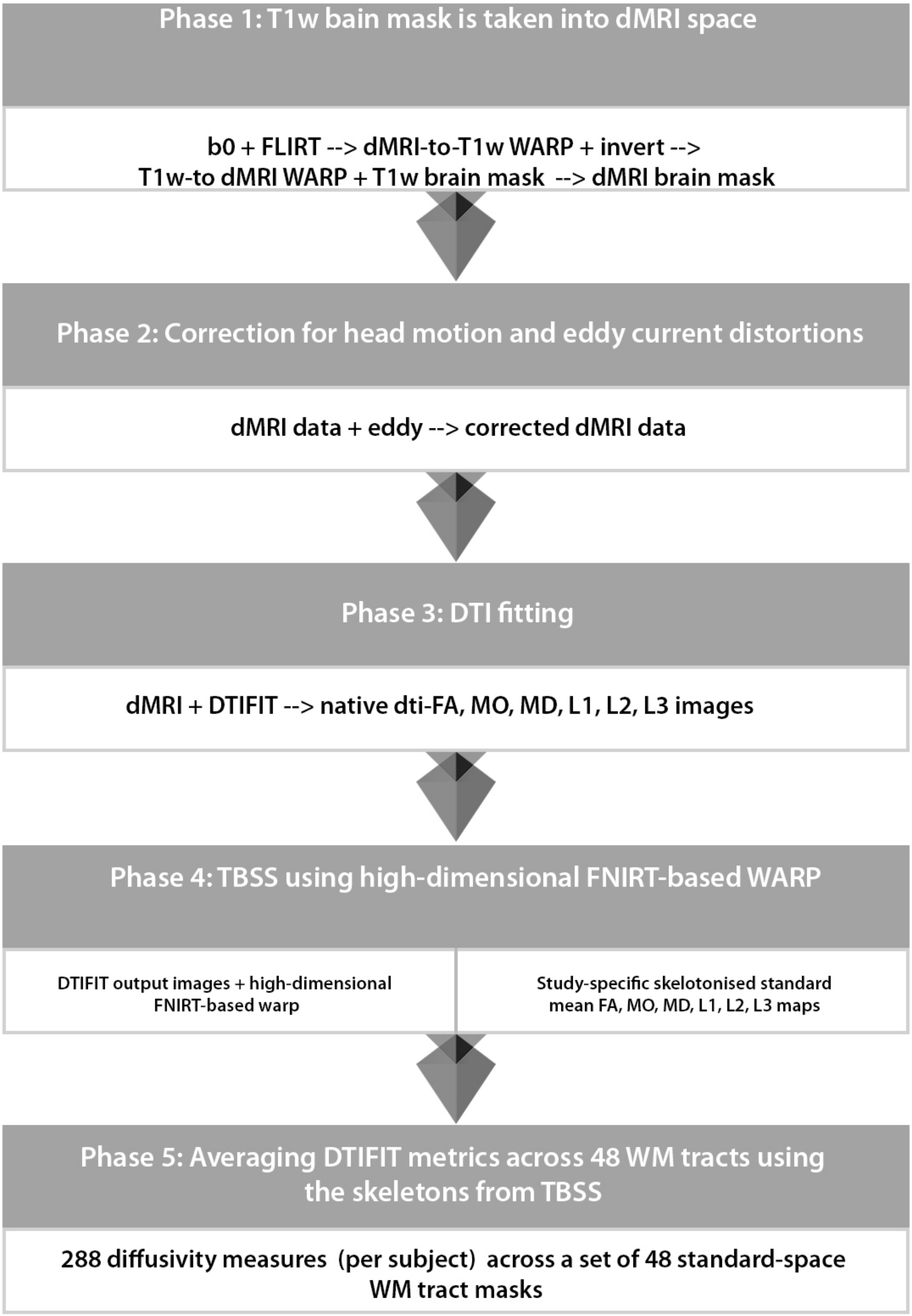

Supplementary Figure 4. General dMRI processing pipeline.

Supplementary Statistical Analysis

Multivariate Associations

We used CCA, a multivariate technique, to seek patterns of covariation between two sets of measures i.e. IDPs and non-IDPs, Figure 5. In essence, we are estimating a "population continuum" of covariation across subjects that jointly characterizes brain imaging and other (non-imaging) data through a single axis. This information is subsequently used to describe each subject's relation to this axis through the value and polarity assigned to their CCA-derived subject weight. That is, each CCA-derived subject weight corresponds to a specific position on the population continuum, and our aim is to characterize the attributes associated with each position.

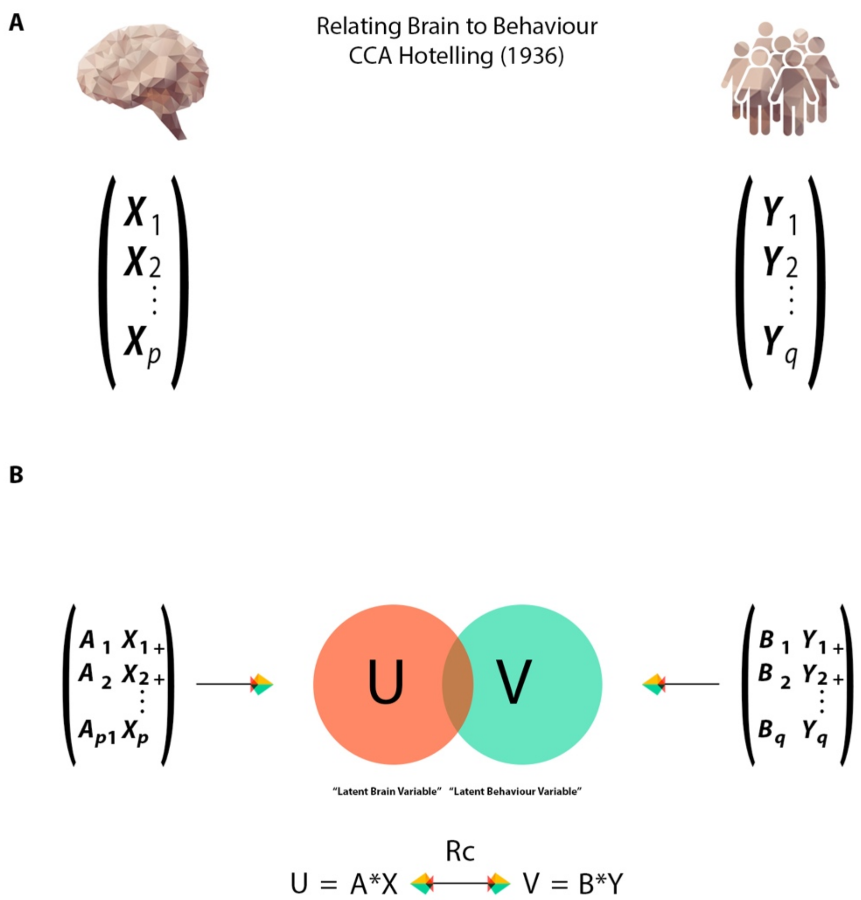

Supplementary Figure 5. Relating brain to behavior using CCA. (A) We used CCA to relate sets of brain-imaging phenotypes, $X_1 \ldots X_p$, (i.e., IDPs) to sets of non-brain imaging phenotypes, $Y_1 \ldots Y_q$, (i.e., non-IDPs). (B) Each CCA-mode identified represents a linear combination of IDPs, 'U', that are maximally correlated to the non-IDPs, 'V'. The strength of this relationship is formally quantified by the canonical correlation coefficient value (i.e., R_c).

Post-hoc Correlations

Supplementary Figure. 6 is a visualization of post-hoc correlations used to relate each original (observed) IDP or non-IDP back to the significant CCA-mode of population covariation. Here we can identify the relative significance of each observed non-IDP or IDP variable in deriving the identified CCA-mode.

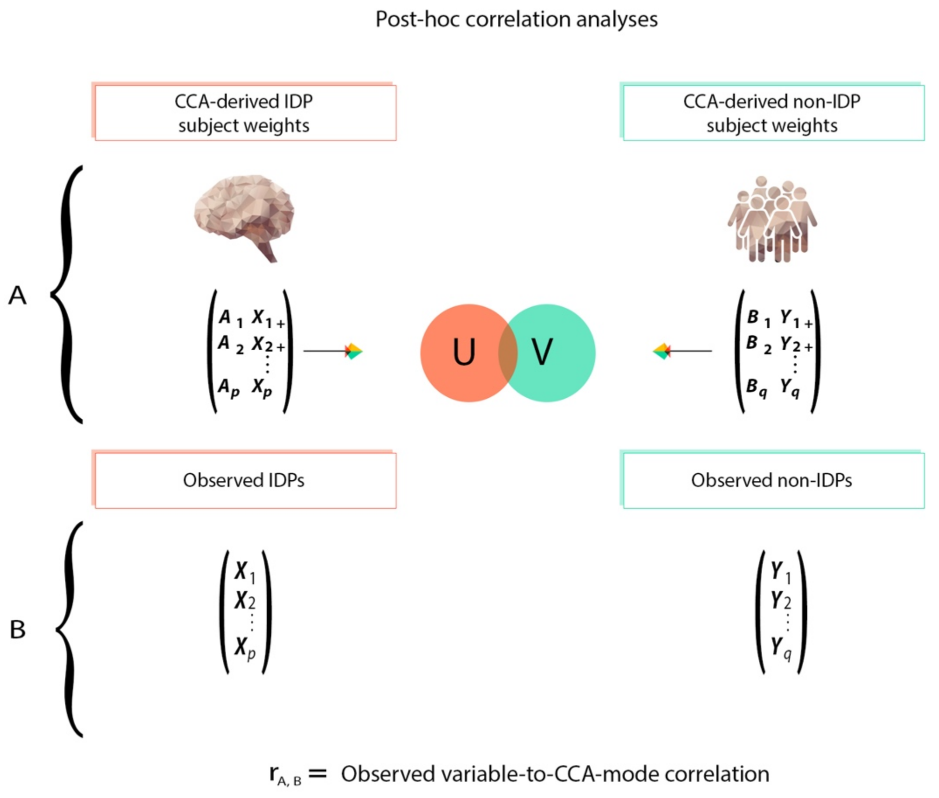

Supplementary Figure 6. Visualization of post-hoc correlation analysis. (A) To relate the estimated CCA-mode, i.e. the UV pair, back to the observed (normalized and deconfounded) variables (B) we use post-hoc correlations, $r_{A,B}$. The correlations computed are interpreted like factor loadings, and simply reflect the amount of variance each observed variable shares with its corresponding canonical variate pair.

Supplementary Results

Study Sample Characteristics of Imaging-Derived Phenotypes by Group

Image-derived Phenotype (IDPs)	GROUP A	GROUP B
	M	*M*
Total brain volume (mm^3)	1483455	1463752
Grey matter volume (mm^3)	756575	747633
White matter volume (mm^3)	726880	716119
Peripheral grey matter (mm^3)	582846	572995
CSF (mm^3)	43769	50203
Thalamus (mm^3)	1105168	1089935
Caudate (mm^3)	1294311	1276844
Putamen (mm^3)	1199740	1183390
Pallidum (mm^3)	1247025	1230117
Hippocampus (mm^3)	1223383	1206753
Amygdala (mm^3)	1235204	1218435
Accumbens (mm^3)	1229293	1212594
Brain stem + 4th ventricle (mm^3)	18887	18197
FLAIR (mm^3)	370	377
FA	0.60445	0.60406
MD	0.00076	0.00077
MO	0.61150	0.61271
L1-L3	0.00076	0.00077

Supplementary Table 1. List and study sample characteristics of image-derived phenotypes (IDP) described by group membership. Total brain volume, volume of individual brain tissues, CSF, volume of subcortical brain structures, and diffusion indices at age ~57 years. Diffusion tensor indices (FA, MD, MO, L1, L2, and L3) are based on eigenvalues ($\lambda 1, \lambda 2, \lambda 3$), which are used to infer on the integrity of white matter microstructure. (Abbreviations: *M* = mean, WMH = white matter hyperintensity, FA = fractional anisotropy, MD = mean diffusivity, MO = mode tensor, L1-L3 = eigenvalues).

Whole-Group Univariate Associations Adjusted for Cognitive Change (CΔ)

A

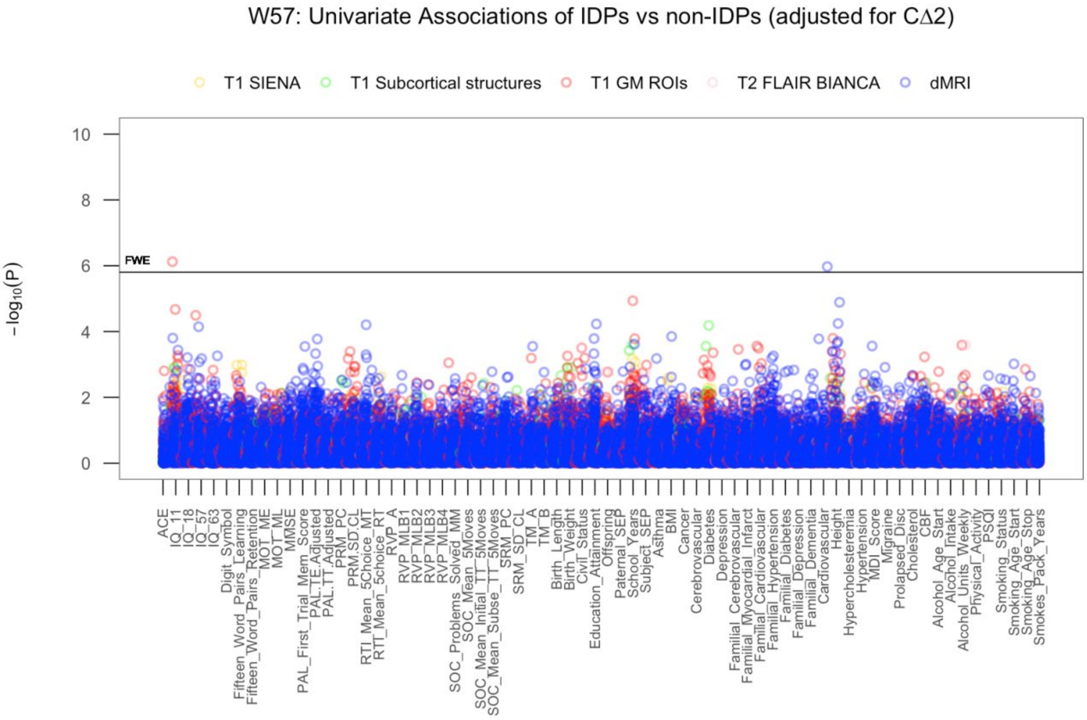

B

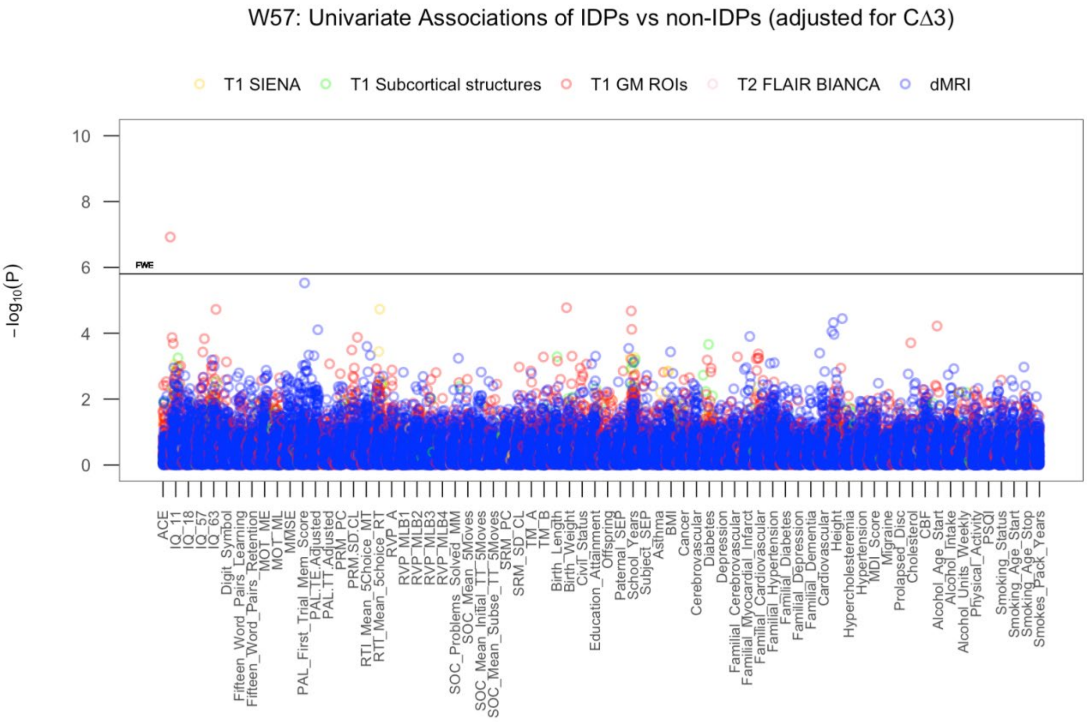

C

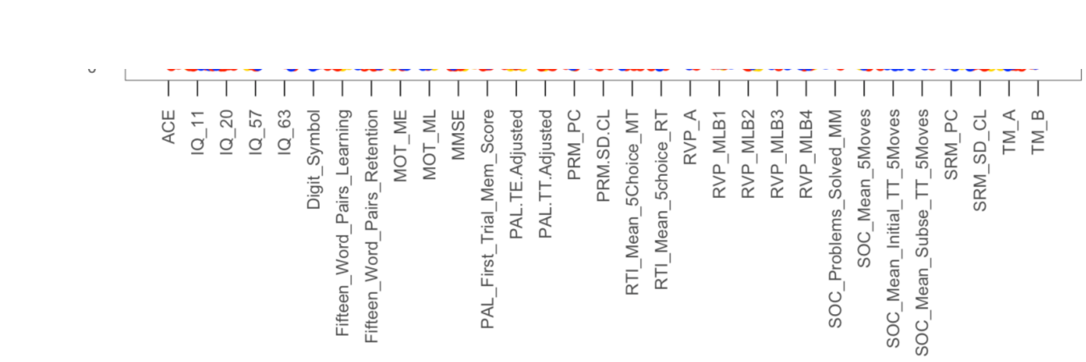

Supplementary Figure 7. The significance of associations between pairwise associations adjusted for cognitive change. Manhattan plots 7A and 7B show all results for 453 IDPs against each of the 70 non-IDPs (31,710 values) adjusted for common confounders (age, motion, head size) and cognitive change variable CΔ2 Figure. 7A (IQ-63-IQ-57), or CΔ3 Figure. 7B (IQ-20-IQ-11). Significance is plotted as -log $_{10}$ p-values, arranged by non-IDPs on the x-axis, multiple testing threshold across all pairwise associations is marked with a black horizontal line (FWE = 5.80 x 10^{-5}). IDPs are distinguished by plotting color to reflect the MRI modality and image processing tool used to estimate each measure. This created five IDP subdomains: T1w-SIENA (yellow), T1w subcortical structures-FIRST (green), T1w GM ROIs-FAST (red), T2w-FLAIR-BIANCA (pink), and dMRI-TBSS (blue). Manhattan plot 7C shows all results for 31 cognitive measures against each of the (other) 39 non-IDPs (1209 values) adjusted for common confounders (age, motion, head size) and cognitive change variable CΔ3 (IQ-20-IQ-11). Multiple testing thresholds across all pairwise associations are marked with a horizontal line, FWE (4.38 x 10^{-4}) top line and FDR bottom line (3.82 x 10^{-3}). All other non-IDPs are defined by plotting color (demographic = yellow, health = red, lifestyle = yellow). (Abbreviations: IDP = image-derived phenotypes, non-IDP = non-image-derived phenotypes, IQ-11, IQ-20, IQ-57, IQ-63 = general intelligence scores at ages ~11, ~20, ~57, and ~63; MOT = motor task; ME = mean error; ML = mean latency; PAL = paired associates learning; TE adjusted = total errors adjusted; TT Adjusted = total trials adjusted; PRM = pattern recognition memory; SD = standard deviation; CL = correct latency; RTI = reaction time task; MT = movement time; RT = reaction time; RVP = rapid visual processing task; MLB1-4 = mean latency block 1 to 4; SOC = Stockings of Cambridge; Mean Initial TT 5 Moves = mean initial total time 5 moves task; Mean Subse TT 5 Moves = mean subsequent thinking time 5 moves task; SRM = spatial recognition memory; TM = trail making task; SEP = social economic position; MDI = Major Depression Inventory; CBF = cerebral blood flow; PSQI = Pittsburgh Sleep Quality Index).

Post-Hoc Correlations

Supplementary Figure 8 is a visualization of post-hoc correlations relating each original (observed) non-IDP with the significant CCA-mode of population covariation. In brief, for each non-IDP (x-axis), the significance of the correlation with the CCA-mode is displayed on the y-axis (r) with each correlation (i.e., individual bar) further accompanied by its corresponding explained variance (r^2). As previously described, all 70 non-IDPs are grouped into four subdomains; cognition, demographic, health and lifestyle. Furthermore, for ease of interpretation, we invert the signs of all non-IDP measures where lower outcome values are indicative of a positive quality or indicator. Thus, when interpreting post-hoc correlations between each non-IDP and the CCA-mode, all positive correlations describe positive contributions to the CCA-mode (e.g., higher cognitive ability, better health status and lifestyle choices), whilst all negative correlations portray unfavorable contributions to the CCA-mode.

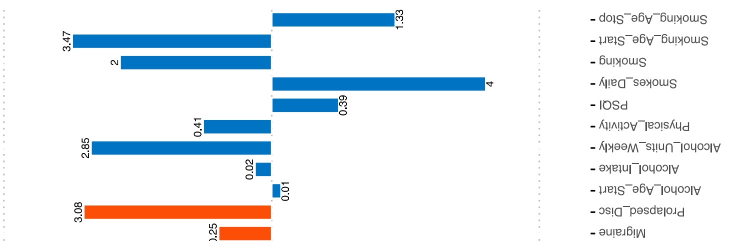

Supplementary Figure 8. A visualization of post-hoc correlation analyses correlating each observed non-IDP to the CCA-mode of population covariation. Correlation values (r) are displayed on the y-axis, and each association is further accompanied by its corresponding percentage variance explained (r^2). This approach illustrates highly influential univariate associations between non-IDPs (x-axis) and the CCA-mode, and thus highlights individual top contributing non-imaging measures of the CCA-mode (i.e., the predictive pattern of measures contributing to the CCA estimations).

a

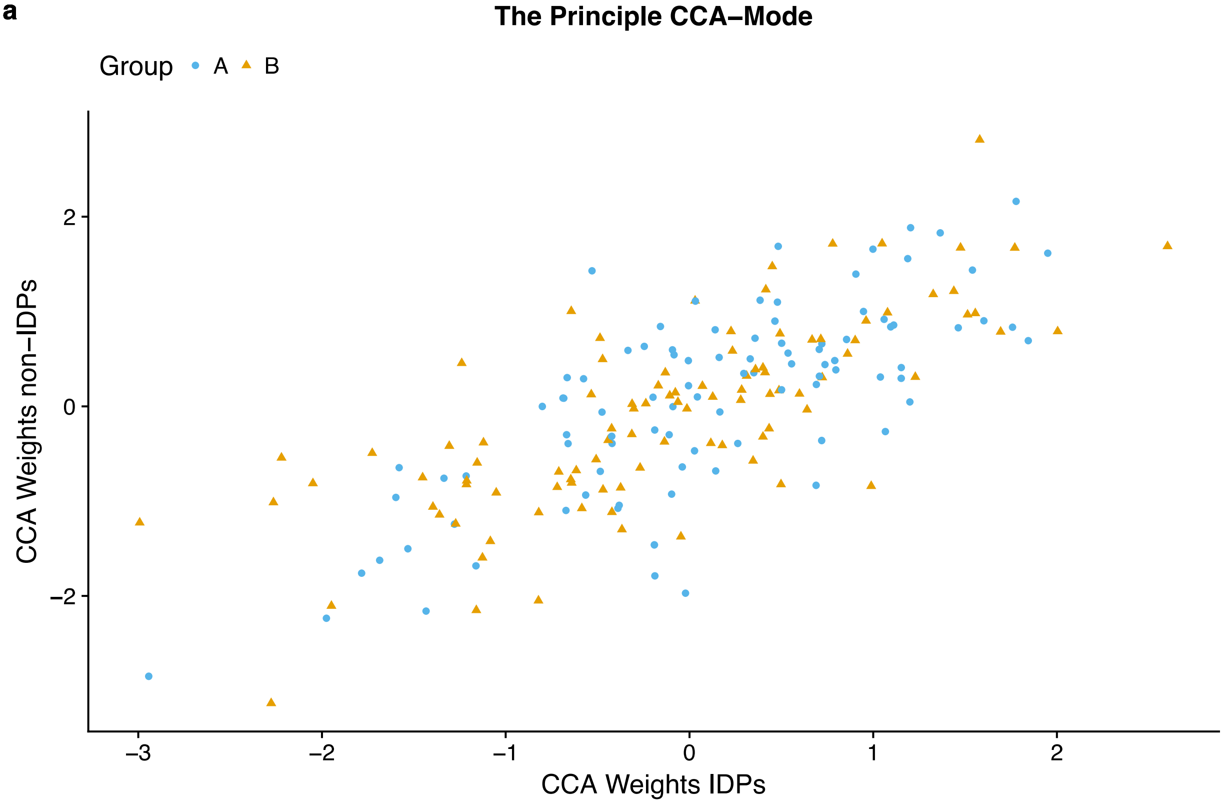

Supplementary Figure 9. Scatter plot of all subject's relationship with the CCA-mode. Group membership indicated by plotting symbol (A=improvers; B=decliners) i.e., individual subject scores in the IDP canonical variate pair (V) vs individual subject scores in the non-IDP canonical variate (U), with one point per subject.

A

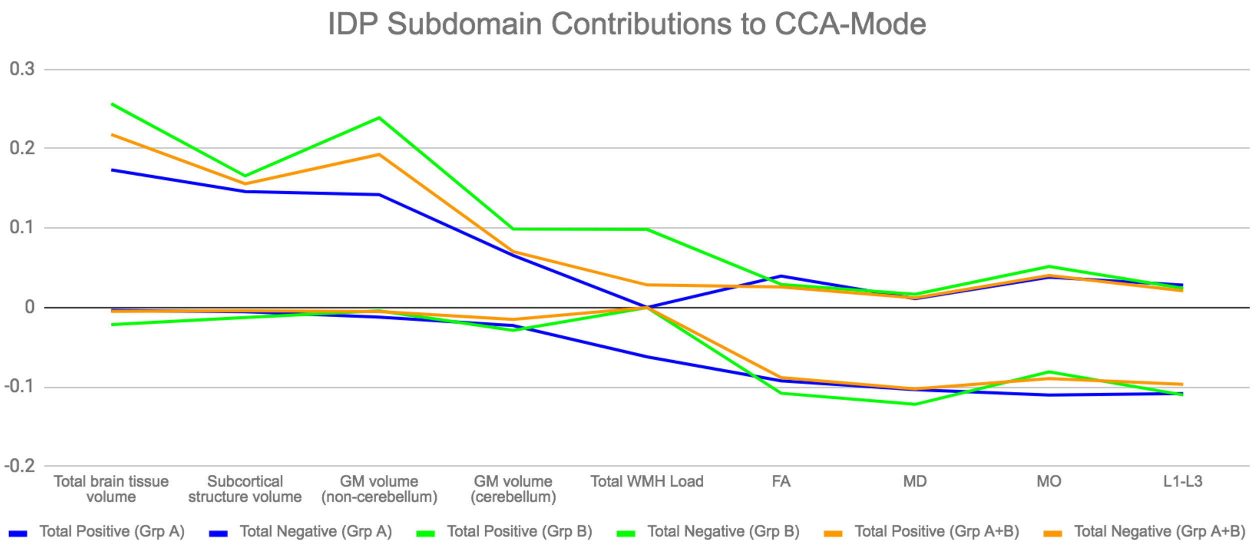

B

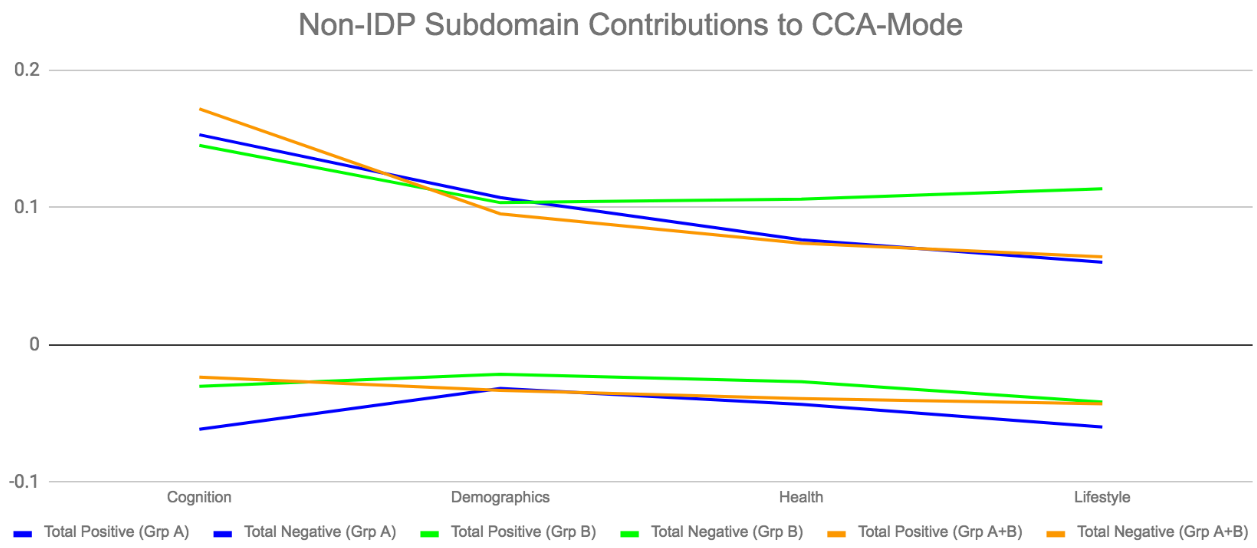

Supplementary Figure 10. Subgroup CCA analysis and post-hoc correlations. Visualization of average subdomain contributions (y-axis = correlation) to the identified CCA-mode computed separately for subgroup A (blue line) and subgroup B (green line) subjects. Results from whole-group analysis is shown in orange for comparison. (Abbreviations: GM = grey matter, WMH = white matter hyperintensity load, FA = fractional anisotropy, MD = mean diffusivity, MO = mode tensor, L1 = 1st eigenvalue, L2 = 2nd eigenvalue, and L3 = 3rd eigenvalue).

Group	Top contributing non-IDPs	r	Top contributing IDPs	r
**Group A ("improvers")**	IQ-57	0.38	MD in splenium of corpus callosum	-0.51
	IQ-20	0.37	L1 in corona radiata (L; R; A; P; S)	-0.49
	MOT (ME)	0.35	MD in corona radiata (L; P)	-0.49
	RTI: 5-choice MT	0.33	ume of GM in precuneous cortex (P)	0.45
	Paternal SEP	0.30	L2 in splenium of corpus callosum	-0.43
	Trail Making B	0.30	L2 in medial lemniscus (L)	-0.43
	SOC: mean initial TT 5-moves	0.26	Total GM + WM volume (unnorm)	0.42
	History of familial dementia	0.26	ume of thalamus (R)	0.40
	IQ-11	0.24	Total peripheral GM (unnorm)	0.39
	IQ-63	0.23	GM volume frontal pole (L)	0.30
	Offspring	-0.21	MD longitudinal fasciculus (R; S)	-0.31
	Education attainment	0.20	GM volume Hippocampus (L)	0.29
**Group B ("decliners")**	Paternal SEP	0.46	FA tapetum (L)	-0.40
	Birth weight	0.42	GM volume supracalcarine cortex (R)	0.39
	Birth length	0.37	FA tapetum (R)	-0.39
	SOC: mean initial TT 5-moves	0.34	MD body of corpus callosum	-0.39
	Familial history of dementia	0.29	GM volume parietal operculum cortex (R)	0.39
	IQ-63	0.28	L1 superior longitudinal fasciculus (R; L)	-0.38
	Trail Making B	0.26	GM volume temporal-occipital gyrus (R)	0.36
	CBF	0.26	MD superior longitudinal fasciculus (R; L)	-0.33
	Trail Making A	0.25	L1 fornix cres+stria termalis (L)	-0.31
	IQ-20	0.25	GM volume in temporal Pole (R;L)	0.30
	IQ-57	0.24	L1 corona radiata (R; S)	-0.26
	IQ-11	0.24	FA limb of internal capsule (R; P)	-0.26
	RTI: 5-choice MT	0.24	GM volume parahippocampal Gyrus (L; AD)	0.23
	15-Word Pairs: Retention	0.22	Total brain GM + WM volume (unnorm)	0.20
	Familial history of Depression	0.20	ume of thalamus (R)	0.22
	Familial history of myocardial infarct	0.20	ume of hippocampus (R)	0.22

Supplementary Table 2. List of top contributing variables pertaining to subgroup CCA analyses. (Abbreviations: MOT (ME) = Motor Task (mean error), RTI: 5-choice MT = Reaction Time: 5-choice movement time, SOC: mean initial TT 5-moves = Stockings of Cambridge mean initial thinking time 5-moves), MD = mean diffusivity, L1 = 1st eigenvalue, L2 = 2nd eigenvalue, GM = grey matter, WM = white matter, L = left, R = right, A = anterior, P = posterior, S = superior, AD = anterior division, unnorm = unnormalised).

Supplementary References

1. Jenkinson, M. et al. Fsl. Neuroimage, 2012. 62: 782-90.
2. Smith, S.M. Fast robust automated brain extraction. Hum Brain Mapp, 2002. 3:143-55.
3. Jenkinson, M. & Smith, S. A global optimisation method for robust affine registration of brain images. Med Image Anal, 2001. 5: 143-56.
4. Jenkinson, M. et al. Improved optimization for the robust and accurate linear registration and motion correction of brain images. Neuroimage, 2002. 17:825-41.
5. Andersson, J.L.R., Jenkinson, M. & Smith, S. Non-linear registration, aka spatial normalisation. 2007.
6. Zhang, Y., Brady, M. & Smith, S. Segmentation of brain MR images through a hidden Markov random field model and the expectation-maximization algorithm. IEEE Trans Med Imag, 2001. 20:45-57.
7. Desikan, R.S. et al. An automated labeling system for subdividing the human cerebral cortex on MRI scans into gyral based regions of interest. Neuroimage, 2006. 31:968-80.
8. Diedrichsen, J. et al. A probabilistic MR atlas of the human cerebellum. Neuroimage, 2009. 46(1): p. 39-46.
9. Patenaude, B. et al. A Bayesian model of shape and appearance for subcortical brain segmentation. Neuroimage, 2011. 56:907-22.
10. Smith, S.M. et al. Accurate, robust, and automated longitudinal and cross-sectional brain change analysis. Neuroimage, 2002. 17:479-89.
11. Smith, S.M. et al. Advances in functional and structural MR image analysis and implementation as FSL. Neuroimage, 2004. 23 Suppl 1: S208-19.
12. Griffanti, L. et al. BIANCA (Brain Intensity AbNormality Classification Algorithm): A new tool for automated segmentation of white matter hyperintensities. Neuroimage, 2016. 141:191-205.
13. Andersson, J.L.R. & Sotiropoulos, S.N. An integrated approach to correction for off-resonance effects and subject movement in diffusion MR imaging. Neuroimage, 2016. 125: 1063-1078.
14. Smith, S.M. et al. Tract-based spatial statistics: voxelwise analysis of multi-subject diffusion data. Neuroimage, 2006. 31:1487-505.
15. Wakana, S. et al. Reproducibility of Quantitative Tractography Methods Applied to Cerebral White Matter. Neuroimage, 2007. 36:630-644.
16. Gunning-Dixon, F.M. et al. Aging of cerebral white matter: a review of MRI findings. Int J Geriatr Psychiatry, 2009. 24:109-17.
17. Jones, D.K. Studying connections in the living human brain with diffusion MRI. Cortex, 2008. 44:936-52.
18. Mori, S. Introduction to diffusion tensor imaging. Elsevier Science & Technology. 2007.
19. Mori, S. & Zhang, J. Principles of diffusion tensor imaging and its applications to basic neuroscience research. Neuron, 2006. 51:527-39.
20. Beaulieu, C. The basis of anisotropic water diffusion in the nervous system - a technical review. NMR Biomed, 2002. 15:435-55.
21. Ennis, D.B. & Kindlmann, G. Orthogonal tensor invariants and the analysis of diffusion tensor magnetic resonance images. Magn Reson Med, 2006. 55:136-46.
22. Sen, P.N. & Basser, P.J. A model for diffusion in white matter in the brain. Biophys J, 2005. 89: 2927-38.
23. Basser, P.J. Inferring microstructural features and the physiological state of tissues from diffusion-weighted images. NMR Biomed, 1995. 8: 333-44.
24. Song, S.-K. et al. Dysmyelination Revealed through MRI as Increased Radial (but Unchanged Axial) Diffusion of Water. NeuroImage, 2002. 17:1429-1436.
25. Burzynska, A.Z. et al. Age-related differences in white matter microstructure: region-specific patterns of diffusivity. Neuroimage, 2010. 49:2104-12.
26. Madden, D.J., Bennett, I.J., & Song, A.W. Cerebral white matter integrity and cognitive aging: contributions from diffusion tensor imaging. Neuropsychol Rev, 2009. 19:415-35.
27. Douaud, G. et al. DTI measures in crossing-fibre areas: increased diffusion anisotropy reveals early white matter alteration in MCI and mild Alzheimer's disease. Neuroimage, 2011. 55:880-90.

28. Sun, S.W. et al. Differential sensitivity of in vivo and ex vivo diffusion tensor imaging to evolving optic nerve injury in mice with retinal ischemia. Neuroimage, 2006. 32:1195-204.

29. Sun, S.W. et al. Selective vulnerability of cerebral white matter in a murine model of multiple sclerosis detected using diffusion tensor imaging. Neurobiol Dis, 2007. 28:30-8.

30. Song, S.K. et al. Diffusion tensor imaging detects and differenciates axon and myelin degeneration in mouse optic nerve after retinal ischemia. Neuroimage, 2003. 20:1714-22.

31. Vernooij, M.W., Arfan, M.I., & Vrooman, H.A. White Matter Microstructural Integrity and Cognitive Function in a General Elderly Population. Arch Gen Psychiatry, 2009. 66.

32. de Groot, M. et al. Improving alignment in Tract-based spatial statistics: evaluation and optimization of image registration. Neuroimage, 2013. 76:400-11.

33. Mori, S. MRI Atlas of Human White Matter. Elsevier Science. 2005:1-276.
